# Integrating an antimicrobial nanocomposite to bioactive electrospun fibers for improved wound dressing materials

**DOI:** 10.1038/s41598-024-75814-2

**Published:** 2024-10-24

**Authors:** Victoria Leonor Reyes-Guzmán, Luis Jesús Villarreal-Gómez, Rubi Vázquez-Mora, Yesica Itzel Méndez-Ramírez, Juan Antonio Paz-González, Arturo Zizumbo-López, Hugo Borbón, Eder Germán Lizarraga-Medina, José Manuel Cornejo-Bravo, Graciela Lizeth Pérez-González, Arturo Sinue Ontiveros-Zepeda, Armando Pérez-Sánchez, Elizabeth Chavira-Martínez, Rafael Huirache-Acuña, Yoxkin Estévez-Martínez

**Affiliations:** 1 Facultad de Ciencias de la Ingeniería y Tecnología, Universidad Autónoma de Baja California, Blvd. Universitario, #1000. Unidad Valle de las Palmas. Tijuana, Baja, Tijuana, CP. 21500 Baja California México; 2 Facultad de Ciencias Química e Ingeniería, Universidad Autónoma de Baja California, Universidad #14418, UABC, Parque Internacional Industrial Tijuana, Tijuana, 22424 Baja California México; 3 Tecnológico Nacional de México, Unidad Tecnológica Acatlán, Campús Acatlán de Osorio, Carretera Acatlán - San Juan Ixcaquistla kilómetro 5.5, Del Maestro, Acatlán, 74949 Puebla México; 4 Tecnológico Nacional de México, Campus Tijuana, Blvd. Alberto Limón Padilla y Av. ITR Tijuana S/N, Colonia Mesa de Otay, Tijuana, C.P. 22500 Baja California México; 5 Centro de Nanociencias y Nanotecnología, Universidad Nacional Autónoma de México, Carr. Tijuana-Ensenada km107, C.I.C.E.S.E, Ensenada, 22860 Baja California México; 6 Facultad de Ciencias de la Ingeniería, Administrativas y Sociales, Universidad Autónoma de Baja California, Blvrd Universidad 1, San Fernando, Tecate, 21460 Baja California México; 7 Instituto de Investigaciones en Materiales, Circuito Exterior S/N Circuito de la Investigación Científica, C.U, Ciudad de México, 04510 México; 8 Facultad de Ingeniería Química, Universidad Michoacana de San Nicolás de Hidalgo, Michoacán, 58060 Morelia Mexico

**Keywords:** Wound dressings, Electrospinning, Chitosan, Silver nanocrystal, Graphene oxide, Materials science, Nanoscience and technology, Drug development

## Abstract

This study investigates the fabrication and characterization of electrospun poly (ε-caprolactone)/poly (vinyl pyrrolidone) (*PCL/PVP*) fibers integrated with a nanocomposite of chitosan, silver nanocrystals, and graphene oxide (*ChAgG*), aimed at developing advanced wound dressing materials. The *ChAgG* nanocomposite, recognized for its antimicrobial and biocompatible properties, was incorporated into *PCL/PVP* fibers through electrospinning techniques. We assessed the resultant fibers’ morphological, physicochemical, and mechanical properties, which exhibited significant enhancements in mechanical strength and demonstrated effective antimicrobial activity against common bacterial pathogens. The findings suggest that the *PCL/PVP-ChAgG* fibers maintain biocompatibility and facilitate controlled therapeutic delivery, positioning them as a promising solution for managing chronic and burn-related wounds. This study underscores the potential of these advanced materials to improve healing outcomes cost-effectively, particularly in settings plagued by high incidences of burn injuries. Further clinical investigations are recommended to explore these innovative fibers’ full potential and real-world applicability.

**Supplementary Information:**

The online version contains supplementary material available at 10.1038/s41598-024-75814-2.

## Introduction

Burn injuries represent a significant public health concern on a global scale, resulting in roughly 265,000 fatalities each year, predominantly concentrated in low- and middle-income nations^1^. These injuries damage cells and blood vessels, disrupting the blood supply essential for wound healing. The complexity of burn wounds, influenced by factors such as oxygen levels, infections, aging, hormones, and nutrition, can significantly interfere with the healing process by affecting the release of growth factors and cytokines^2^. Conventional treatments, including various dressings and skin substitutes, aim to facilitate wound healing by maintaining a moist environment, offering protection, and alleviating pain^3^. However, these traditional solutions, such as bandages and gauze, often fall short of effectively managing highly exudative or infected wounds, leading to a need for more advanced and effective solutions.

Building upon the foundational research documented^4^; this study seeks to bridge the gap between basic research and practical application. Here, we explore the integration of the *ChAgG* nanocomposite within *PCL/PVP*electrospun fibers, aiming to enhance the functionality of wound dressings by leveraging the nanocomposite’s antimicrobial and biocompatible properties^5^.

Electrospun nanofibers, particularly those combining the mechanical and biological benefits of *PCL/PVP* with *ChAgG*, offer promising enhancements over traditional dressings. These fibers can be engineered to release therapeutic agents directly at the wound site, providing targeted antimicrobial action and supporting the healing environment without frequent dressing changes. The biocompatibility and effectiveness of such fibrous scaffolds have been demonstrated in their ability to support fibroblast growth and combat a range of pathogens including Gram-negative and Gram-positive bacteria and fungi^5^.

The *ChAgG* nanocomposite in *PCL/PVP*fibers enhances wound healing by combining the benefits of its components^4^. Graphene Oxide (*GO*) improves mechanical strength, making the dressing more durable and promoting cell attachment and growth by offering a high surface area. It also disrupts bacterial membranes, reducing infection risk^6^. Silver nanocrystals provide a sustained release of silver ions, which kill bacteria and reduce inflammation, creating a better healing environment^7^. Chitosan promotes blood clotting, cell attachment, and tissue regeneration while maintaining a moist wound environment and adding antimicrobial properties^8^. The *PCL/PVP*matrix is a biodegradable scaffold that steadily releases therapeutic agents, reducing the need for frequent dressing changes and supporting consistent tissue repair^5,9^. Overall, this combination promotes regeneration, prevents infection, and maintains a balanced environment for optimal healing.

*PCL/PVP-ChAgG*fibers can offer several advantages over other advanced wound dressings due to their combination of antimicrobial efficacy, biocompatibility, strength, and customization^5,9,10^. Silver nanocrystals, graphene oxide, and chitosan provide a potent and sustained antimicrobial effect, reducing the risk of antibiotic resistance, unlike dressings that rely on a single agent^4^. These fibers also promote fibroblast proliferation and tissue regeneration, making them more effective than synthetic dressings focusing only on protection^3^. Graphene oxide enhances the mechanical strength and durability of the fibers, which are less fragile than hydrogels or biopolymers. Additionally, they allow for the controlled release of therapeutic agents, reducing dressing changes and ensuring consistent healing^11^. Their ability to be engineered for specific wound types further sets them apart from traditional dressings like gauze, making them highly adaptable and versatile for complex wounds^12^.

Hence, the integration of chitosan, silver nanocrystals, and graphene oxide (*ChAgG*) into *PCL/PVP*electrospun fibers will significantly enhance wound healing by providing sustained antimicrobial activity, promoting fibroblast proliferation, and maintaining mechanical durability^13^; this will lead to improved healing outcomes in complex wounds, such as burn injuries when compared to conventional wound dressings.

We anticipate that the *PCL/PVP-ChAgG* fibers will exhibit superior antibacterial efficacy, particularly against common wound pathogens while promoting cell proliferation and tissue regeneration. Additionally, the mechanical properties of the fibers are expected to ensure durability and flexibility, reducing the need for frequent dressing changes. These factors combined should demonstrate the potential of these advanced wound dressings to accelerate healing and reduce complications, particularly in chronic and burn-related wounds.

Hence, this research aims to advance the development and characterization of *PCL/PVP-ChAgG* fibers, examining their morphological, physicochemical, and mechanical attributes to gauge their suitability for advanced wound healing applications. By advancing the *ChAgG* nanocomposite’s application from a primary antimicrobial agent to a functional component of electrospun fibers, this research addresses the pressing need for more effective dressings to manage the complexities of chronic and burn-related wounds. Through a comprehensive evaluation of these innovative fibers, we hope to provide a scientifically sound basis for their future clinical development and potential to improve healing outcomes in a cost-effective manner.

## Materials and methods

### Materials

Poly (ε-caprolactone) (PCL) with an average molecular weight of 80,000 g/mol, purity of 99.9% for general laboratory use (sourced from Sigma-Aldrich), and poly (vinyl pyrrolidone) (PVP) with an average molecular weight of 40,000 g/mol, with purity of 99% (also obtained from Sigma-Aldrich) were utilized in this study. The solvents employed were tetrahydrofuran (THF) with a molecular weight of 84.93 g/mol (supplied by Tecsiquim) (purity of ≥ 99%) and dichloromethane (DCM) with a molecular weight of 72.11 g/mol (procured from Sigma-Aldrich) and purity of ≥ 99.8%, ACS reagent grade or HPLC grade. The antimicrobial nanocomposite, *ChAgG*, was prepared using chitosan (Ch) obtained from Merck, with a purity of ≥ 85%. Graphene powders (Gr) were sourced from LP Bond Research and Development of the Third Millennium S.A. of C.V, and AgNO_3_ from OMNICHEM was used as received. Solvents for this process included acetic acid at a 1% v/v concentration and hydrogen peroxide (H_2_O_2_) (purity of ≥ 99%) at 30% v/v, both from Merck. To synthesize the silver nanocrystals (AgNCs), AgNO_3_ from the commercial supplier OMNICHEM with a purity of ≥ 99% was utilized. Ammonium hydroxide (NH_4_OH) (purity of ≥ 99% for analytical applications), distilled water, *Allium sativum* extract (garlic) was acquired from the central market of the City of Acatlán de Osorio, México.

### Synthesis of ChAgG nanocomposite

The *ChAgG*nanocomposite was synthesized per the methodology detailed in our prior work by Estevez-Martínez et al^4^., and the patent application for the synthesis of *AgNCs*^14^. In these experiments, garlic was finely crushed in bi-distilled water at a concentration of 10.9% w/v for 30 s using a 600-Watt food processor. The resulting mixture was left to stand for 12 h, during which it changed from yellowish to turquoise blue; this procedure facilitated the extraction of sulfur compounds from the garlic, including alliin, (+)-S-methyl-L-cysteine sulfoxide, and γ-L-glutamyl-S-allyl-L-cysteine^15^. The solid organic remnants were vacuum-filtrated through a cloth mesh following this timeframe. A reflux setup comprising a heating and stirring apparatus, flask, and refrigerant was employed to heat the garlic solution to 75 °C. Upon reaching this temperature, 1 mmol of AgNO_3_was introduced and left to react for 30 min. The solution transitioned from turquoise blue to brick-brown, signaling the generation of silver nanocrystals^16^. After 30 min of reflux reaction, oxidized graphene was combined with the solution of silver nanocrystals (AgNCs) at a mass ratio of 2:1, maintaining the temperature at 75 °C for another 30 min. Following this, a chitosan solution was introduced at a concentration of 2% w/v, with an initial concentration of 0.6% v/v for the entire solution, and the reaction temperature was maintained at 75 °C for the final 30 min. Finally, a filtration setup consisting of a Kitasato flask, Büchner funnel, and a nitrocellulose membrane with a 0.20 μm diameter was utilized to collect the resulting solids, comprising the graphene nanocomposite, silver nanocrystals, and chitosan, forming the *ChAgG* compound.

For instance, the temperature of 75 °C was likely selected to facilitate the successful reduction of silver ions and the formation of nanocrystals while ensuring that the chitosan and graphene oxide components remain stable. Similarly, maintaining this temperature during the introduction of chitosan likely helped promote the proper integration of the components without degrading their functional properties. The reaction durations, such as 30 min for the silver nanocrystal formation and subsequent chitosan addition, have been optimized to achieve a balance between effective nanoparticle synthesis and preventing overexposure, which could lead to aggregation or reduced activity^4,14,17^.

On the other hand, the choice of the 2:1 mass ratio of oxidized graphene to silver nanocrystals is a crucial aspect of the synthesis process that likely balances the synergistic effects of both components while optimizing the composite’s antimicrobial efficacy and mechanical properties. This ratio was selected based on the need to ensure that graphene oxide, which provides mechanical strength and surface area for cell attachment, is present in sufficient quantities to support the silver nanocrystals and prevent their agglomeration. The larger proportion of oxidized graphene helps disperse the silver nanoparticles more evenly, enhancing their interaction with bacterial cells without overwhelming the polymer matrix or causing cytotoxicity^18^.

Moreover, graphene oxide’s ability to generate reactive oxygen species (ROS) can work in tandem with the silver ion release from the nanocrystals, resulting in a more potent antibacterial effect. The 2:1 ratio may have been optimized to maximize this antibacterial synergy while minimizing potential adverse effects, such as excessive cytotoxicity from too much silver. This specific ratio have been derived from prior optimization studies or literature references, where varying proportions of graphene and silver were tested to find an effective balance between mechanical reinforcement, biocompatibility, and antimicrobial action^4,19^.

### Electrospun fibers preparation (PCL/PVP and PCL/PVP-ChAgG fibers)

#### Preparations of matrix solution

Three *PCL/PVP* solutions were prepared with different percentages of *ChAgG* antimicrobial compound at 1, 5, and 10%. The amount of each polymer needed to prepare each *PCL/PVP* blend is 85:15 w/w using Tetrahydrofuran (*THF*) and Dichloromethane (*DCM*) as solvents. The mixture is placed on the heating plate with constant stirring at 200 rpm and a temperature of 50 °C for 24 h until a homogeneous mixture is obtained.

#### Electrospinning

After preparing a solution with a final polymer concentration of 13% w/v, 5 mL was loaded into a 5 mL plastic syringe with a 12 mm blunt tip needle. The syringe was then securely attached and stabilized in an injection pump programmed to operate at a flow rate of 0.5 mL/h. The process was carried out with a voltage of 20 kV applied, maintaining a distance of 20 cm between the needle’s tip and the collector. All these procedures were conducted at room temperature within an environment with a relative humidity level ranging from 16 to 24%. The ambient conditions were regulated and controlled within a sealed acrylic chamber, utilizing temperature and humidity sensors and desiccants, such as silica gel, to maintain stability (Figure S1).

### *ChAgG* composite characterization

#### X-ray powder diffraction (XRD) (ChAgG nanocomposite)

X-ray Diffraction (XRD) is essential for determining the crystalline structure of the ChAgG nanocomposite within PCL/PVP fibers. XRD provides information on graphene oxide’s phase composition and crystallinity, silver nanocrystals, and chitosan. Identifying characteristic diffraction patterns helps confirm the successful incorporation of these components into the fibers. Additionally, XRD can detect changes in crystallinity caused by the integration of the nanocomposite, offering insights into how these structural features impact the material’s mechanical properties, stability, and overall performance in wound healing applications. In this case, the reagent’s purity and silver’s crystalline structure were confirmed via powder X-ray diffraction (XRD) utilizing a Bruker AXS Model D-B Advance. The nanocrystals, integrated into the garlic, underwent exposure to a temperature of 95 °C for 8 days in an oven (FELISA brand, maintained within approximately ± 5 °C) to aid in solvent and water evaporation. Subsequently; this analysis was performed as there were no observed concerns regarding the detachment of the nanocrystals from the crystallizers, even under standard laboratory temperature and humidity conditions.

#### X-ray photoelectron spectrometry (XPS) (ChAgG nanocomposite)

X-ray Photoelectron Spectroscopy (XPS) is critical to analyzing the ChAgG nanocomposite, providing detailed data on its elemental composition and chemical states. XPS detects elements like silver, carbon, oxygen, and nitrogen, confirming the incorporation of silver nanocrystals, graphene oxide, and chitosan. It reveals surface chemistry, including the oxidation state of silver (Ag⁰ vs. Ag⁺) and chemical bonds between the components, offering insights into their molecular interactions; this is essential for understanding the nanocomposite’s antimicrobial properties and chemical stability in wound healing applications. The XPS equipment has a µ-FOCUS 500 X-ray monochromator, which operates using the *ChAgG* excitation line. Reference points were established based on the adventitious C1s, O1s, and Ag peaks to determine binding energies. Background subtraction was conducted using a Shirley baseline, and core-level spectra were fitted using mixed Gaussian/Lorentzian functions.

#### Raman spectroscopy (ChAgG nanocomposite)

Raman spectroscopy is particularly significant for this investigation as it offers detailed information about the molecular vibrations and structural characteristics of the graphene oxide and chitosan components in the *ChAgG* nanocomposite. It is crucial for detecting the G-band and D-band of graphene, which correspond to the graphitic structure and defects in the material. Additionally, Raman can reveal how these components interact with silver nanocrystals and confirm the successful integration of the nanocomposite by identifying shifts or changes in the spectral peaks; this provides essential data on the chemical structure and potential functional interactions within the nanocomposite. The Raman spectrometer had a 532 nm laser and a CCD detector. The detector’s temperature was kept below 5 °C to ensure optimal performance, and a laser filter with a setting of 0.3 was utilized. Measurements were taken using an x50 objective, resulting in a magnification of the image size by 1.4 × 100. The confocal analysis was executed with a spatial resolution of 1 × 1 μm, a dimension well within the size range of a graphite particle (which measures 10 μm in the material under investigation).

#### High-resolution transmission electron microscopy (HRTEM) (ChAgG nanocomposite)

The structural characteristics of the nanocrystals and size within garlic (*Allium sativum*) were examined using High-Resolution Transmission Electron Microscopy (HRTEM) utilizing a JEOL JEM-1200EX instrument. For the SEM analysis, garlic (*Allium sativum)* samples had to undergo solvent evaporation in an oven (FELISA brand, ± 5 °C) at 95 °C for 5 days, after which the *AgNCs* were extracted from their containers (crystallizers). In the case of the High-Resolution Transmission Electron Microscopy (HRTEM) analysis, garlic samples were subjected to the same procedures as in the previous tests. However, the temperature was elevated to 400 °C and maintained for 3 days to ensure more thorough solvent evaporation. To prepare the samples for analysis, a solution containing silver nanoclusters in methanol was created by agitating in an ultrasound bath.

### Electrospun fibers characterization (PCL/PVP and PCL/PVP-ChAgG fibers)

#### Fourier transforms infrared spectroscopy (FTIR) (PCL/PVP and PCL/PVP-ChAgG fibers)

The fibers underwent infrared spectroscopy analysis to investigate potential chemical changes induced by the material processing through electrospinning and to identify the presence of Cs and Ns. Specimens measuring 0.5 × 0.5 cm were taken from each scaffold and placed on the lens of the ATR module. These samples were then analyzed over a spectrum ranging from 4000 to 400 cm^−1^ wavenumbers. The resulting spectra were further analyzed using Omnic software.

#### Thermogravimetric analysis (TGA) and Differential scanning calorimetry (DSC) (PCL/PVP and PCL/PVP-ChAgG fibers)

Thermogravimetric Analysis (TGA) and Differential Scanning Calorimetry (DSC) assess the thermal properties and stability of the *ChAgG* nanocomposite in *PCL/PVP* fibers. TGA measures weight loss with temperature to determine thermal stability and decomposition, identifying degradation points for chitosan, PCL, and PVP, as well as residual materials like graphene oxide and silver nanocrystals. DSC analyzes heat flow to detect transitions like melting and crystallization, revealing how the nanocomposite’s components affect thermal behavior, it confirms its suitability for biomedical uses such as wound dressings where thermal stability is crucial.

For thermogravimetric analysis, 5 mg of samples were placed in platinum trays and subjected to heating. The heating process began at room temperature (around 25 °C) and continued up to 600 °C at a heating rate of 10 °C/min, all under a nitrogen atmosphere. In the case of the differential scanning calorimetry, samples were loaded into platinum trays, compressed, and tightly sealed. These samples underwent the same conditions as the thermogravimetric analysis. The resulting thermograms were analyzed using TA universal analysis software.

Conversely, evaluating silver nanocrystals utilized a high-resolution TA Instruments 2950 thermogravimetric analyzer. The samples were examined over a temperature range starting from room temperature and extending to 500 °C at a heating rate of 10 °C per minute while exposed to air. For this analysis, only 2 mL of each sample in liquid form was utilized without prior evaporation, as reliable results could be obtained in the liquid state. The liquid samples were stored in a refrigerator until the analysis was conducted.

#### Scanning electron microscopy (SEM) (PCL/PVP and PCL/PVP-ChAgG fibers)

Scanning Electron Microscopy (SEM) is critical for examining the surface morphology and microstructure of the *ChAgG* nanocomposite within *PCL/PVP* fibers. SEM provides high-resolution images that reveal the fiber diameter, surface texture, and overall uniformity of the electrospun fibers. It helps assess the distribution of chitosan, silver nanocrystals, and graphene oxide throughout the fibers, ensuring even dispersion, essential for consistent antimicrobial and healing properties. Additionally, SEM can detect any surface defects or irregularities, offering insights into the structural integrity and suitability of the material for wound dressing applications. The JEOL JSM 7600 F microscope from JEOL Ltd. in Tokyo, Japan, was used. A voltage of 20 kV was applied to capture the images, and a gold coating was employed. The diameter and porosity of the selected nanofibers were quantified using Image J software; this entailed taking 30 measurements from at least two distinct images of each sample, which were magnified to 4,000X and 6,000X. Energy dispersive X-ray spectroscopy (EDX) was performed by analyzing the SEM images at a magnification of 500X; this was conducted with Pd metallization of 4.0 nm and a 10 keV beam energy, while the accelerating voltage was set at 200 kV.

#### X-ray tomography (PCL/PVP and PCL/PVP-ChAgG fibers)

X-ray tomography is a non-destructive imaging technique that generates detailed 3D views of the internal structure of materials. The *ChAgG* nanocomposite in *PCL/PVP* fibers reveals key aspects like fiber morphology, porosity, and pore size, which are essential for assessing oxygen permeability and fluid absorption for wound healing. It also detects defects, ensures mechanical stability, and confirms the uniform distribution of chitosan, graphene oxide, and silver nanocrystals, crucial for consistent antimicrobial action and healing; this technique provides a comprehensive understanding of the fibers’ structural features and role in wound healing. A digital representation of the *PCL/PVP-ChAgG* electrospun fibers was generated using an X-ray tomography-based method. The reconstruction process involved slice acquisition and subsequent image processing. The airflow direction was aligned with the z-axis, while the surface of the membrane was defined as parallel to the x-y plane. Slice acquisition was performed using an RX Solution Easy Tom XL, tomography instrument equipped with X-ray sources at both 160 kV and 230 kV. In this study, a 40 kV tube generated the X-ray cone beam, with the tube current set at 160 µA. Raw X-ray digital images were captured using a high-resolution and high-speed CCD detector. The distance between the X-ray source and the specimen was 7.109 mm, distance between the X-ray source and the detector was 610.65 mm. The imager operated at a frame rate of 1 fps. After filtration with a Tukey window, 1352 slices were obtained in the vertical (x-z) plane, each consisting of 1845 × 1845 voxels. The adequate voxel size was 1.48 μm. Subsequently, these slices were imported into the ImportGeo-Vol module within the GeoDict software package for 3D processing.

#### Mechanical testing (PCL/PVP and PCL/PVP-ChAgG fibers)

Mechanical testing assesses the strength, flexibility, and durability of *PCL/PVP-ChAgG* fibers, ensuring they withstand typical wound dressing stresses. Critical tests include tensile strength, which evaluates the force needed to break the fibers, and elastic modulus, measuring stiffness and deformation under stress. A balanced modulus ensures flexibility while maintaining strength. Elongation at break gauges the fibers’ ability to stretch before breaking, which is essential for adapting to body movements. Mechanical testing confirms that the fibers balance strength and flexibility for adequate wound protection and comfort. The fibers were cut into rectangular specimens measuring 3 cm in length and 1 cm in width using scissors, and the thickness of the electrospun polymer samples was measured at various points using a Vernier caliper. Data on the mechanical properties of the fibers were collected using the EDMS-FG V4.6.2 software (NidecShimpo). Specimens were subjected to tensile loads until they fractured, utilizing an FG-3005 dynamometer with a load capacity of 50 N and a resolution of 0.005 N; this dynamometer was mounted on the CHATILLON MT 500 H test bench column. All samples were positioned between two grips separated by a distance of 1 cm. The tensile test was conducted at an 8 mm/min rate, performed at room temperature (21 °C) following the ASTM D1708 standard. From the tensile tests and by the ASTM D638 standard, the elastic modulus, tensile strength, and elongation at break were calculated. Each experiment was performed with three replicates, as shown in Figure S2.

####  Antibacterial test (PCL/PVP and PCL/PVP-ChAgG fibers)

Circular fiber samples, each with a diameter of 0.6 cm², were prepared using a hole punch. Both sides of these samples underwent sterilization by exposure to ultraviolet (UV) light for 15 min. Subsequently, these sterilized fiber samples were placed at the bottom of individual wells within a 96-well microplate. Bacterial strains, including *Staphylococcus aureus* (ATCC 23235) and *Escherichia coli* (ATCC 25922), were cultured in previously sterilized Muller-Hinton medium at 35 °C for 24 h. After preparing the bacterial cultures, 200 µL of each inoculum, suspended in fresh medium (*E. coli* and *S. aureus*), was added to the respective wells, containing a fiber sample. A negative control was included alongside the test samples, comprising fibers made from PCL/PVP 85:15 without any antimicrobial agents. Phenol and penicillin, each at a concentration of 10 mg/mL, were used as positive controls. All bacterial cells that encountered the fibers were incubated at 35 °C for 24, 48, and 72 h. After the incubation period, the fibers were removed, and the solution was analyzed using a microplate reader (Thermo Scientific) at a wavelength of 620 nm.

####  MTT cell viability test (PCL/PVP and PCL/PVP-ChAgG fibers)

The samples underwent sterilization by being exposed to ultraviolet (UV) light for 15 min on both sides within a laminar flow hood. They were immersed in DMEM (Dulbecco’s Modified Eagle Medium) for one day. The ATCC CCL-1 fibroblast cell line NCTC Clone 929, derived from *mus musculus* tissue, was employed for cell culture. These cells were cultured in DMEM supplemented with 10% fetal bovine serum (FBS) and 100 U/mL penicillin-streptomycin at 37 °C in an atmosphere containing 5% CO_2_ until they reached a confluency of 80%. The scaffolds were introduced into tissue culture microplates using a direct contact method, adhering to the ISO-10993-5 guideline. Specifically, 10,000 L-929 fibroblast cells were exposed to each well’s *PCL/PVP-ChAgG* scaffolds for 24 h under the same incubation conditions. A positive control involving diluted phenol at a concentration of 10 mg/mL was utilized, while the negative control consisted of a cell suspension without any treatment. Following incubation, 10 µL of MTT (3-(4,5-dimethylthiazol-2-yl)-2,5-diphenyltetrazolium bromide) solution was added to 100 µL of medium in each well. The plate was gently tapped on the side to ensure thorough mixing and then incubated at 37 °C for 4 h. Subsequently, 200 µL of DMSO (dimethyl sulfoxide) was added directly into each well medium and pipetted several times to dissolve the formazan salt. Finally, the absorbance signal was measured using a 570/630 nm spectrophotometer. These experiments were conducted in triplicate, and the average absorbance and standard deviation were calculated.

## Results and discussions

### ChAgG nanocomposite characterization

#### X-ray powder diffraction (XRD) (ChAgG nanocomposite)

Figure S3A of the XRD data depicts the presence of *ChAgG*, where the d values of 5.8300, 4.3700, 4.1700, and 4.1700 correspond to chitosan characteristics with PDF 00-039-1894. These values correspond to four reflections (indicated by the blue lines in Figure S3B) with 100% intensity, corresponding to (hkl) planes: (120), (102), (022), and (200). Additionally, the XRD data in red lines reveal the presence of carbon, identified through PDF 00-003-0401, with d values of 3.4000, 2.0600, and 1.6800. As anticipated, AgNCs are not detected in the XRD data, as they constitute less than 3% of the material. It is well-known that XRD can detect materials present in the 1 to 3% range, depending on the specific material under examination. Furthermore, within the diffractogram analysis, the amorphous portion, which includes the nanostructured organic material and the sample holder, is observed as a comprehensive reflection, as expected.

Examining the diffractogram of the *ChAgG* nanocomposite from a different perspective in Figure S3B, the blue lines represent chitosan characteristics with PDF 00-039-1894. Additionally, the red lines indicate the presence of carbon, identified through PDF 00-003-0401.

#### X-ray photoelectron spectrometry (XPS) (ChAgG nanocomposite)

X-ray Photoelectron Spectroscopy (XPS) was employed to detect the presence of AgNCs in the *ChAgG* nanocomposite in conjunction with carbon and oxygen. Reference XPS spectra were also acquired for graphene (Gr) and oxidized graphene (GrOx). The binding energy range for carbon (C1s) extended from 295 to 281 eV, while for oxygen (O1s), it ranged from 540 to 528 eV. The silver signal fell within the range of 364 to 376 eV. The analysis covered all three energy ranges for the *ChAgG* nanocomposite, whereas the reference materials (Gr and GrOx) were analyzed solely for C1s and O1s.

The deconvolution of the C1s and O1s peaks was performed based on established data from various reports on XPS, including those authored by^20–23^. In the case of Gr and GrOx (as illustrated in Figure S6A), the C1s peak emerged at 284.5 eV, indicating sp2 hybridization, at 285.6 eV for sp3 hybridization, at 286.6 eV for oxidized graphene featuring hydroxyl groups (–OH), at 287.6 eV for carbonyl (C = O), at 288.6 eV for carboxyl groups (–COOH) typical of oxidized graphene, at 290 eV for carbonate (C–O*–C = O), and 290.9 eV for pi-pi* (π-π*) electronic transitions specific to graphene sheets. The O1s peaks were observed at 531.2, 532.1, 533.4, 534.3, and 535.5 eV, representing molecules of carboxyl (-COOH), carbonyl (O = C), hydroxyl (O–C), carbonate (C–O*–C = O), and water (H_2_O), respectively.

The presented data exhibits the peaks of the nanocomposite *ChAgG*(as depicted in Figure S6B). In the C1s region, the deconvoluted bands resemble those found in graphene (Gr), comparing the presence of characteristic graphene peaks in C1s within the nanocomposite. However, the O1s band encompasses the silver-oxygen peaks falling in the range of 528 to 535 eV, as previously investigated since the late 1990s^24^. and still referenced today^25^. Specifically, it shows peaks at 530.4, 530.9, 531.6, and 532.4 eV, representing ionic bonds (O-Ag), covalent bonds (Ag-O), hydroxyl (O-C), and carbonate (C-O*-C = O), respectively. Two distinctive peaks related to the presence of AgNCs are also evident^25,26^, with binding energies for Ag3d_5/2_ at 367 eV and Ag3d_3/2_ at 373 eV, as confirmed by prior studies.

An examination of the percentage distribution of peaks within C1s for both samples (as shown in Figure S6) indicates a notable decrease in sp2 hybridization in the nanocomposite, approaching 20% compared to the Gr sample. However, when graphene is oxidized (GrOx), this value increases by approximately 2%, presumably due to the slight exfoliation during oxidation^23^. Furthermore, there is an increase in the hybridization of defects (sp3), -OH, and carboxyl groups compared to the reference samples^27^. The rise in π-π* electronic transitions (at 290.9 eV) in oxidized graphene compared to pristine graphene suggests sheet exfoliation, possibly induced by peroxide^28^.

Concerning the O1s peak of the *ChAgG*nanocomposite, around 50% is ascribed to ionic interactions between oxygen and silver, while 21% corresponds to covalent interactions between these atoms; this indicates that the nanocrystals are connected to the oxidized graphene and chitosan molecules through both of these mechanisms^25,29^.

In this study, Raman and XPS analyses of chitosan and *AgNCs* were excluded, as the main aim was to verify the presence of silver and its interaction with oxygen atoms, whether from oxidized graphene or chitosan. Such evidence has been extensively documented.

In summary, the study effectively characterized a nanocomposite consisting of graphene, chitosan, and silver nanocrystals (*ChAgG*) through diverse structural characterizations (XRD, TEM, XPS) and spectroscopic analyses (Raman).

#### Raman spectroscopy (ChAgG nanocomposite)

Raman spectroscopy, a crucial tool in identifying graphene-based materials, reveals strong and distinctive bands at 1580 and 1362 cm^−1^, corresponding to the G band and the disorder-induced D band, respectively. These bands, resembling those observed in graphite, are not solely governed by phonon properties but also influenced by the electronic traits of the materials. For instance, in single-layer graphene (1LG), the linear band structure and the configurations of various bands play a significant role. The generation of harmonic and combining modes, like the 2D modes, defines a triple resonance Raman scattering (TRRS) process, linked with the linear dispersion of its electronic bands. The presence of the 2D band allows for the differentiation of multilayer graphene, which typically appears around ∼2700 cm^−1 30 ^.

The G band at 1580 cm^−1^ corresponds to vibrations within the graphite plane and is characterized by phonon modes in the E2g symmetry (longitudinal optics (LO) and transverse optics (TO) in the plane). Consequently, the position of the G mode, referred to as Pos(G), is sensitive to external perturbations such as defects, doping, strain, and temperature. Therefore, it is commonly used to investigate how graphene-based materials and related devices react to external factors. In the high-frequency region, due to the weak interlayer coupling in multilayer graphene (MLG), the intrinsic N-layer graphene (NLG) typically has a Pos(G) of nearly 1582 cm^−1^, and it is not very sensitive to the number of graphene layers (N). However, graphene structures with fewer than ten layers exhibit parameters that significantly depend on the number of layers (N).

The D band at 1362 cm^−1^is primarily associated with vibrational modes of the sp2 rings. Consequently, this band is linked to the various disorders that NLGs may have, displaying spectral characteristics similar to those of the 2D mode. The fundamental modes of the 2D and D peaks necessitate a surface defect for their activation in Raman scattering. Therefore, the absence of the 2D band can indicate the presence of high-quality monolayer graphene (1LG)^31^ (Figure S5).

Based on the results and the tabulated values presented in Figure S5, it was observed that the obtained spectra enable the identification of signals associated with the characteristics of graphene nanostructures, as described in the work by^32^. Furthermore, to discern the distinctive bands of chitosan and silver nanocrystals, their positions were ascertained using information from the literature. Chitosan bands typically occur at 2885 cm^−1^ and 1654 cm^−1^, with the amine group of chitosan appearing at 1593 cm^−1^, as reported by^33^ and ^34^, respectively.

In the case of silver nanocrystals (AgNCs), as previously reported, these nanostructures, when integrated into chitosan aerogel, exhibit significant interactions in the range of 1000 to 1800 cm^−1^, as documented by^35^; this interaction accounts for the substantial broadening noticed in the *ChAgG*spectrum, where the signals from graphene, chitosan, and silver nanocrystals coincide; this broadening phenomenon has similarly been documented in instances where silver nanocrystals interact with graphene bilayers, as detailed in the study by^36^.

The symmetrical shape of the signal identified as 2D implies the presence of a graphene monolayer, and the 2D/G signal ratio indicates that graphene lacks significant structural defects until it interacts with AgNCs. However, the increased D/G ratio indicates the presence of surface defects caused by oxidation and a disruption in the lamellar structure when graphene interacts with silver nanocrystals and chitosan. In the oxidized state, these oxides are located at the edges of the graphene sheets, facilitating bonding between the silver nanocrystals and the graphene sheets. Chitosan, a crucial stabilizer in this interaction, contributes to the stability of these two nanostructures^37^.

#### High-resolution transmission electron microscopy (HRTEM) (ChAgG nanocomposite)

Estevez-Martinez and colleagues (2023) utilized high-resolution transmission electron microscopy (HRTEM) to observe and document the morphology of metallic silver nanocrystals^4^. Figure S4 presents the outcomes of their synthesis process involving 1 mmol *AgNO*_*3*_ in polycrystals weighing 0.0425 mg, revealing rectangular nanocrystals with approximate dimensions of ~ 90 × 60 nm (Figure S4).

The HRTEM image provided insights into the identification of crystalline phases, space groups, and orientations of the nanoparticles (NPs) concerning the lattice fringes’ d-spacing; this analysis was essential, particularly in light of the X-ray diffraction (XRD) results that elucidated the crystalline structure of silver nanocrystals through AgCl minerals like chlorargyrite, as discussed in the preceding section.

Within the same batch of nanocrystals, a significant discovery emerged—arrangements aligned with the crystalline properties of super crystals (3D assemblies), including face-centered cubic (fcc), hexagonal close-packed (hcp), body-centered cubic (bcc) structures, and even random compact packaging (rcp). These diverse structures emanate from collective properties, driven either by dipolar interactions or intrinsic order, by established literature^38,39^.

Expanding on the discoveries made by Estevez-Martinez et al., a fresh *HRTEM* micrograph presents the outcome of amalgamating *AgNCs* with chitosan and graphene, culminating in the formation of a novel composite: chitosan-silver nanocrystals-graphene (*ChAgG*); this visual representation intricately showcases the silver nanocrystals (*AgNCs*) embedded within chitosan layers, all nestled within a chitosan matrix. Upon closer examination, the inter-fringe separation between neighboring *AgNCs* measures around 0.235 nm and 0.2439 nm; notably, *HRTEM* micrographs highlighted well-defined lattice fringes, particularly in the d111Ag plane, affirming the crystalline nature of the prepared *AgNCs*^40–42^. Nonetheless, the electron diffraction pattern encounters some distortion owing to the interference of chitosan molecules with electron diffraction; this additional perspective not only enriches our understanding of the *ChAgG* composite’s structural nuances but also offers insights into the interplay between *AgNCs* and the chitosan matrix.

A separate study by Khan et al. (2019) harnessed *HRTEM*for sizing crystalline nanocrystals, recording dimensions of 12.6 ± 3.8 nm—twice the size observed in our investigation; this sizing assessment, consistent with face-centered cubic (fcc) crystallography, was validated by powder X-ray diffraction (XRD) and selected area electron diffraction (SAED)^43^.

Likewise, Chung et al. (2016) explored the green synthesis of *AgNCs* from plant extracts, utilizing *HRTEM* and scanning electron microscopy (*SEM)* for morphological observations. Comparable to our research and Khan’s findings, they employed *XRD*for crystallographic analysis, with particle sizes ranging from 1 to 100 nm^44^.

Comparative analysis with reported literature revealed smaller particle sizes with agglomerated cubic structures in *AgNCs*synthesized from plants. The resultant nanostructured material demonstrated exceptional bioactive properties^44,45^.

### PCL/PVP-ChAgG fibers characterization

#### Fourier transforms infrared spectroscopy (FTIR)

All samples were analyzed by Fourier Transform Infrared Spectroscopy (FTIR) to obtain evidence of the *ChAgG* incorporation into *PCL/PVP* fibers (Figure S7, Table 1).

**Table 1:** Functional group analysis of the *PCL/PVP* and *PCL/PVP-ChAgG* electrospun fibers.

Functional groups	PCL/PVP signals (cm^−1^)	PCL/PVP ChAgG 1% signals (cm^−1^)	PCL/PVP ChAgG 5% signals (cm^−1^)	PCL/PVP ChAgG 10% signals (cm^−1^)
**a) C-H**	2945.60	2947.00	2947.99	2950.50
	2865.84	2867.81	2866.66	2868.78
**b) C = O**	1722.03	1722.66	1722.58	1722.74
**d) C = O**	No signal	1661.34	1677.14	No signal
**c) C-N**	1238.58	1238.42	1238.99	1238.25
	1164.28	1164.27	1164.50	1164.65
**e) C-O-C**	1107.16	1107.36	1108.34	1107.13

Thus, a characteristic *PCL* sharp peaks at 1722 cm^−1^ were observed in all membranes corresponding to the stretching vibration of the b ) C = O and at 1107 cm^−1^could be identified as ester group e) C-O-C due to the stretching vibration of the signal, coinciding with the literature^46^, however group e) C-O-C also belongs to the solvent dichloromethane (*DCM*).

Regarding PVP, characteristic signals were also found at 1164–1239 cm^−1^ due to the stretching of the amide group c) C-N and the stretching peak at 1780 cm^−1^ of the primary amine N-H, similar, which indicates that the electrospinning process of the *PCL/PVP*membranes was carried out successfully^47^. On the other hand, peaks at 960 cm^−1^ were also observed, which is associated with the ring vibration of the *PVP*compound; this last signal was present in all the samples according to Versei^46^, which is indicative of the *PCL/PVP* relation, confirming the presence of both polymer in the electrospun samples.

On the other hand, we can also observe the peaks of 1661–1677 cm^−1^ corresponding to the stretching vibration of the amide group d) C = O that indicates the presence of the chitosan compound (Ch), although according to studies shown in the literature, the characteristic chitosan are close at 3400 cm^−1^ (aliphatic primary amine) and 2800 cm^−1^peaks^48^. In the *PCL/PVP-ChAgG* 10% solution, it is impossible to observe this peak’s presence. For the presence of the silver compound (Ag), something similar happened. In the literature, Ag nanoparticle spectrometry was performed and peaks of 1647 cm^−1^were found, indicating the presence of the amide group in the nanoparticles formed^49^, corresponding to our peak of 1667 cm^−1^ and that the control does not present the same as the sample of 10% d) C = O. Aliphatic amines were also found around 1162–2139 cm^−1^ c) C-N. The peaks mentioned above confirm the presence of *PCL* and *PVP* polymers. The bands at 2866–2950 cm^−1^ produced by the stretching vibration of the a) C-H group indicate the presence of tetrahydrofuran, one of the solvents used.

Regarding the vibrations of chitosan, as mentioned above, studies shown in the literature indicated that the characteristic peaks of chitosan are approximately 3400 cm^−1^ (aliphatic primary amine) and 2800 cm^−1^; however, we observed peaks of 1661–1677 cm^−1^that are attributed to the stretching vibration of the N-acetyl group of the bond d) C = O that indicates the presence of the compound Ch^48,50,51^.

When *ChAgG* is added, we see a displacement of the group b) C = O of the *PCL/PVP* bands from 1722 cm^−1^ to 1661 cm^−1^, which indicates that this is where the *PCL/PVP* joined with the *ChAgG*compound. We see similar behaviors in the literature^52,53^, fabricated *PCL/PVP-AgNP* nanofibers where after adding AgNO_3_ to the solution, the *PVP* absorption band at 1658 cm^−1^ shifted to 1663 cm^−1^, indicating the presence of interaction between Ag and the C = O group; these vibrations are present in the nanofibers with the antibacterial compound, however, in the *PCL/PVP-ChAgG* 10% sample this signal is not perceived, which tells us that something happened, Still, it should be clarified that this test is not decisive to affirm the presence of the *ChAgG* compound.

#### Thermogravimetric analysis (TGA) and differential scanning calorimetry (DSC) (PCL/PVP and PCL/PVP-ChAgG fibers)

In Figure S8 of TGA and DSC, the percentage of mass of 90% from 100 ºC begins a process of mass loss, which is visualized in the curve of Figure S8; we can conclude that it is because of the degradation of the organic matter present in the samples, since from that temperature the water begins to evaporate, like the *NH*_*4*_*OH*.

Figure S8 reveals a promising outcome-a substantial yield of silver nanocrystals from garlic. This exciting result paves the way for the potential use of these silver nanocrystals in practical applications.

On the other hand, an essential characteristic of a wound dressing system is the thermal stability of the polymeric fibers because burn injuries can reach temperatures up to 41 °C^54,55^. As per the literature, the PCL’s reported glass transition temperature of PCL is approximately − 60 °C, whereas its melting point is 60 °C^54,55^., coinciding with our results. In the case of PVP, its melting point is around 150–180 °C^56^. The thermograms depicted in Figure S9 indicate that PCL/PVP with 85:15 ratios exhibit a melting point of approximately 45.89 °C, aligning with the previously mentioned literature. The gradual decrease in melting point confirms the presence of PVP in the polymeric blend, with higher PVP content correlating with lower melting points. The melting points observed for the polymers investigated in this study are consistent with those reported in existing literature^54,56,57^, determined as suitable for practical applications; these thermograms indicate that the inclusion of *ChAgG* does not lead to a significant alteration in the melting point, providing a considerable temperature margin (over 10 °C) above the temperature associated with burn injuries (41 °C).

Regarding the degradation temperature and the initial temperature at which 5% mass loss was observed, a slight decrease can be noted in all samples containing *ChAgG* compared to the control fibers. Subsequently, at the critical degradation temperature where 50% mass loss occurred, a more dispersed variation in temperature was observed, approximately at 410 °C. Notably, when comparing the total degradation temperature between the unloaded *PCL/PVP* fibers and the *PCL/PVP-ChAgG* fibers, a significant difference is evident, with a higher proportion of *ChAgG* leading to more residual components that were not decomposed at approximately 780 °C, contrasting with the *PCL/PVP* control, which underwent complete degradation at 556 °C. These findings underscore the presence of the *ChAgG* blend in the *PCL/PVP* fibers (Table 2).

**Table 2:** Important temperatures in TGA-DSC analysis of the *PCL/PVP* and *PCL/PVP-ChAgG* electrospun fibers.

Sample	Glass transition temperature (^o^C)	Melting temperature (^o^C)	Initial degradation (^o^C)	Critical degradation (^o^C)	Total degradation (^o^C)
***PCL/PVP***_**control**_	− 63.63	45.89	367.27	411.80	555.77
***PCL/PVP*** ***ChAgG*** **1%**	-63.23	48.42	351.19	408.58	780.47
***PCL/PVP*** ***ChAgG*** **5%**	-63.72	51.79	354.86	414.87	779.22
***PCL/PVP*** ***ChAgG*** **10%**	-62.96	51.27	367.43	412.11	779.89

ND, Not defined.

#### Scanning electron microscopy (SEM) (PCL/PVP and PCL/PVP-ChAgG fibers)

All *PCL/PVP-ChAgG* samples were successfully electrospun under the conditions used, and all samples developed fibrous structures with no artifacts and bulbs; because of the proposed application, fibrous were intended to be thick to improve their mechanical properties and handleability. *PCL/PVP* control fiber has an average fiber diameter of 2.9 ± 1.7 μm, and it was observed that with the addition of the *ChAgG* to the samples, all fibers were slimmed down. In the case of *PCL/PVP-ChAgG* 1%, the average fiber diameter was 1.5 ± 0.5 μm, for *PCL/PVP-ChAgG*5% 2.0 ± 0.7 μm, and for *PCL/PVP-ChAgG*10% 0.6 ± 0.3 μm, respectively. They were the thicker *PCL/PVP* control fibers and *PCL/PVP-ChAgG* 10% with the most decreased diameters. Except for the *PCL/PVP-ChAgG* 10% fibers, all sample distribution was more abundant at 1.5 μm. Moreover, the EDX filter was used to find Ag on the fibrous samples and evidence of the presence of the *ChAgG* formulations (Figure S10). Fortunately, all loaded samples were encountered with an Ag signal, and no presence was found in the *PCL/PVP* control fibers (Table 3).

**Table 3:** Measurement of the fiber diameters with the ImageJ program and EDX analysis. Ž: average. SD: standard deviation.

Sample	Ž± SD (µm)	Ag (wt %)	C (wt %)	O (wt %)
***PCL/PVP***_**control**_	2.9 ± 1.7	0	74.93	25.07
***PCL/PVP-ChAgG*** **1%**	1.5 ± 0.5	0.01	76.12	23.87
***PCL/PVP-ChAgG*** **5%**	2.0 ± 0.7	0.06	65.39	34.56
***PCL/PVP-ChAgG*** **10%**	0.6 ± 0.3	**0.24**	78.95	20.81

In a study by Jaganathan^58^, electrospun nanofibers were fabricated using a polyurethane and cobalt nitrate composite, with a fiber diameter measuring 0.6 ± 0.2 μm. In comparison, pristine polyurethane fibers had a larger diameter of 1.10 ± 0.2 μm. Interestingly, it was observed that the fibrous mats composed of PU/cobalt nitrate exhibited thinner fibers when compared to those made of pure PU; this reduction in fiber diameter in the electrospun PU/cobalt nitrate composite resulted from the dilution of the polymer concentration due to the addition of cobalt nitrate to the polyurethane matrix; this decrease in fiber diameter, similar to our findings, is advantageous as it may promote improved adhesion and proliferation of fibroblasts, contributing to the growth of new skin tissue.

Jaganathan^58^, reported electrospun nanofibers of poly urethane/cobalt nitrate composite with a fiber diameter of 0.6 ± 0.2 μm and poly (urethane) pristine fibers of 1.10 ± 0.2 μm. It was noted that the study found that the fibrous mats made from the PU/cobalt nitrate composite exhibited thinner fibers compared to those composed of pure PU; this decrease in fiber diameter in the electrospun PU/cobalt nitrate composite was attributed to the lowering of polymer concentration resulting from the inclusion of cobalt nitrate into the polyurethane matrix. These thinner fibers were observed to be even smaller in diameter than those in the pristine PU material, which is potentially beneficial for promoting improved adhesion and proliferation of fibroblasts, thus supporting the growth of new skin tissue. This potential for improved tissue growth is a hopeful prospect for future research and applications.

Williams^59^, prepared electrospun fibers of poly (ethylene glycol) (PEO) using the coaxial electrospinning to be proposed as wound dressings. These fibers were loaded with cerium (III) nitrate and were randomly aligned with fiber diameters of 1.2 ± 0.2 μm and 1.1 ± 0.3 μm. Release studies demonstrated that the PEO-base dressings deliver ultimately their content after the first hour with no cytotoxicity effects; the authors discussed that these fibers with these diameters are effective for this specific application. The fiber diameters obtained from that study are similar and intended for the same application.

Finally, Zhao, Y-T., 2020^60^, reported the design and configuration of a self-powered portable melt electrospinning device for the fabrication of in situ wound dressings of poly (lactic acid) (PLA), poly (lactic-co-glycolic acid) (PLGA 75:25), and polycaprolactone (*PCL*) in animal models, where the final fiber´s diameters are around 27.5 μm, 21.2 μm and 35.8 μm, respectively. The authors concluded that these wound dressings are effective systems. As can be observed, these last diameters are significantly thicker than our fibrous systems, confirming their potential use in this application.

#### X-ray tomography (PCL/PVP and PCL/PVP-ChAgG fibers)

**Figure 1:**
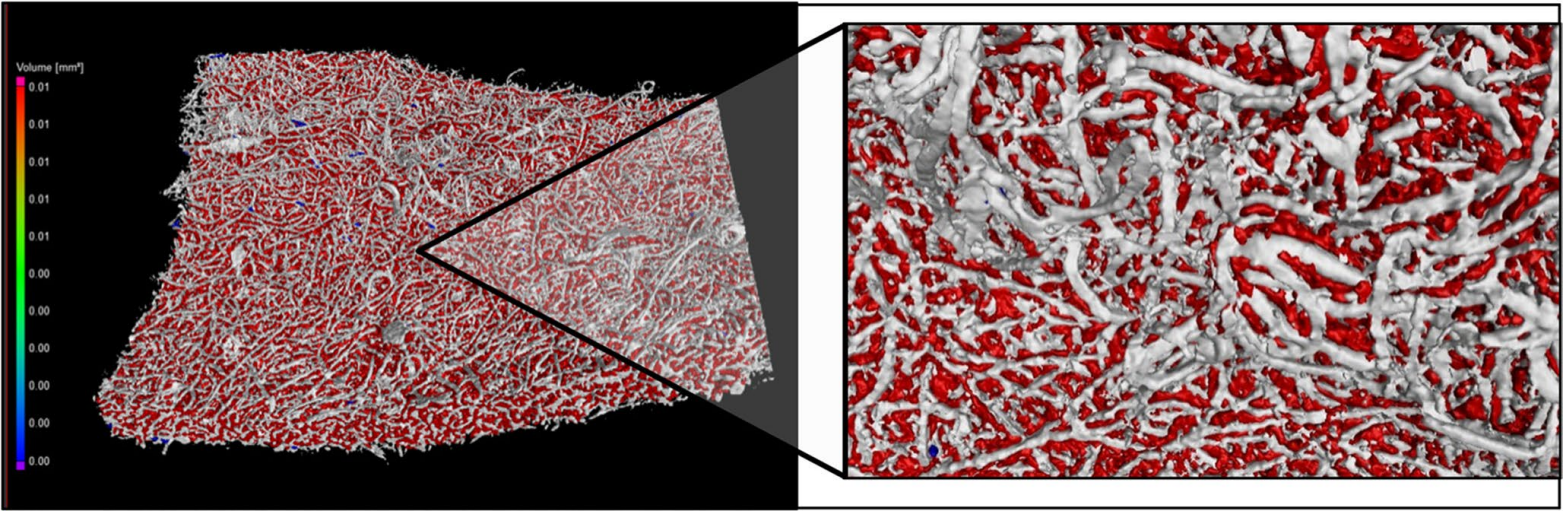
Electrospun fibers in white and de air surrounded in red. Images were developed using software VGSTUDIO MAX (Version 2022.4) (https://www.volumegraphics.com/en/products/vgsm.html).

X-ray tomography, a form of non-destructive testing (NDT), is an appealing technique for analyzing high-density objects because it can differentiate between various materials and structures present within a single object, such as electrospun fibers. Several factors can affect the performance of the CT process, including the surface extraction technique employed. In this study, the surface extraction technique relies on defining a gray level value as a similarity reference, enabling the identification of both the fibers and the surrounding air; this is crucial for analysis, as membranes made from such electrospun fibers need to allow for oxygenation to facilitate a more effective re-epithelialization process. Figure 1 displays a high-definition image of a CT scan conducted on the electrospun fibers. In the image, the color white represents the fibers, while the color red represents the air between them, constituting 24% of the total volume of the object; this air percentage within the object allows for oxygenation of the injured area, thereby promoting improved re-epithelialization.

**Figure 2:**
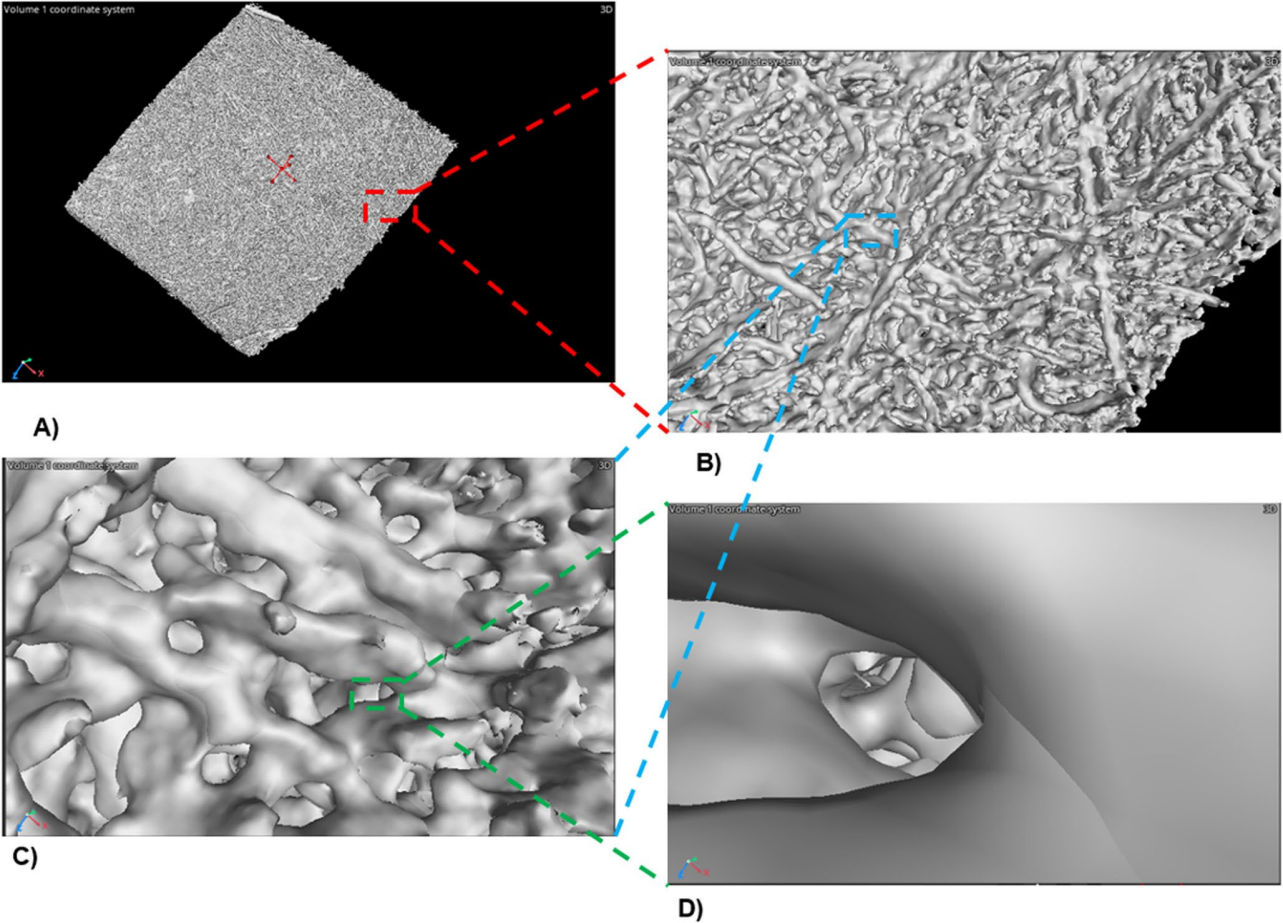
3D volume from X-ray tomography of PCL/PVP and PCL/PVP-ChAgG fibers. Analysis of interconnected pores. Images were developed using software VGSTUDIO MAX (Version 2022.4) (https://www.volumegraphics.com/en/products/vgsm.html).

X-ray tomography is also very useful for generating a 3D volume of the electrospun fibers to analyze the interconnection of the fibers and the defects in an accurate membrane model. In Fig. 2A, a complete 3D model of the membrane is shown. Figure 2B, C, and D show the interconnection of the fibers in detail and some pores among them.

#### Mechanical testing (PCL/PVP and PCL/PVP-ChAgG fibers)

**Figure 3:**
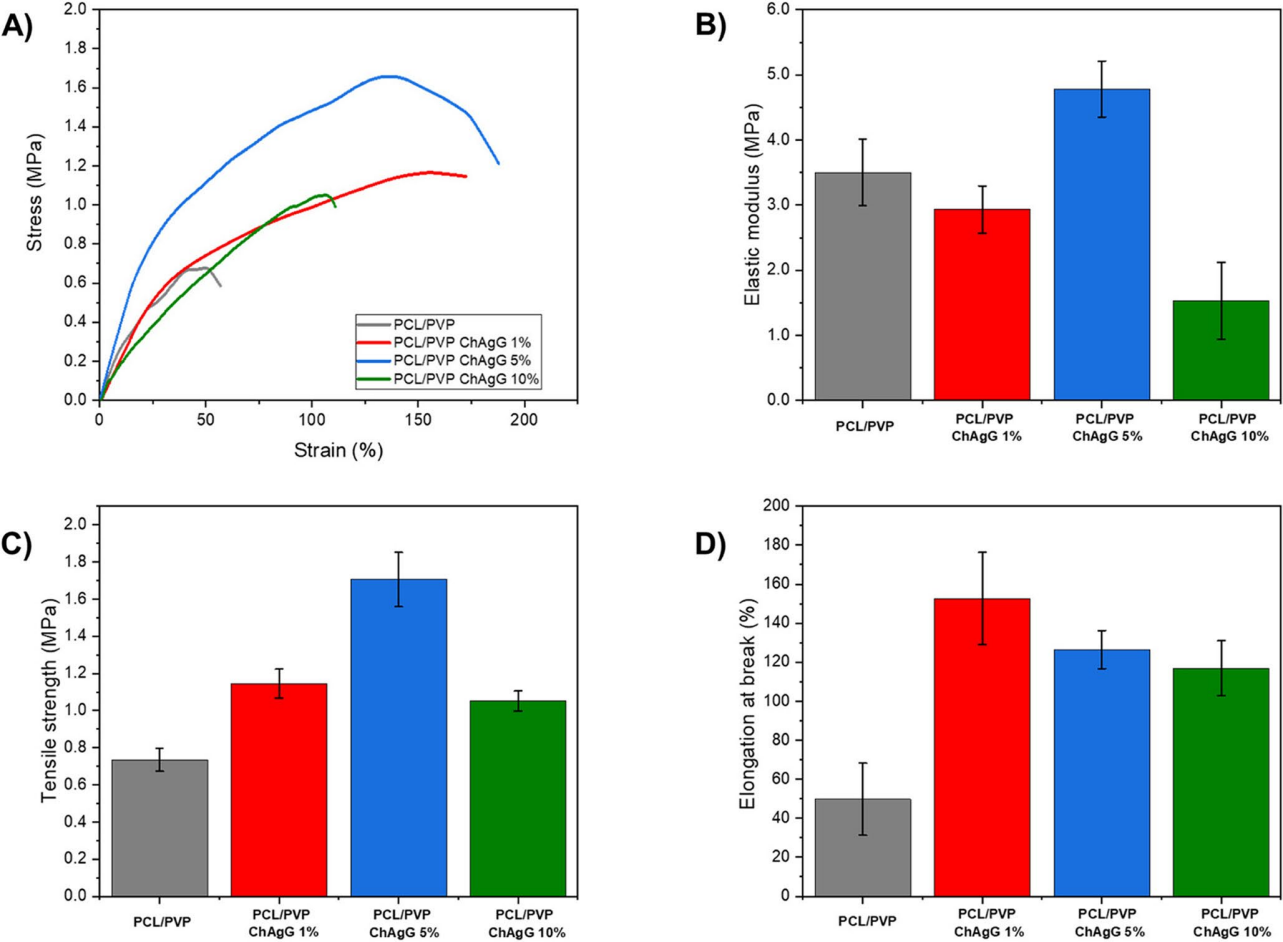
Mechanical behavior of *PCL/PVP* and *PCL/PVP-ChAgG* fibers. (**A**) Stress-strain curves. (**B**) Elastic modulus. (**C**) Tensile strength. (**D**) Elongation at the break.

The mechanical properties of the electrospinning films were evaluated and the tensile test results are shown in Fig. 3. In our study, specimens around 3 cm x 1 cm were used, which are commonly used in tensile testing of nanofibrous materials, especially for applications such as wound dressings; this size ensures uniform stress distribution during mechanical testing and allows for reproducibility across tests. The dimensions are also sufficient to fit standard testing equipment, as seen in prior studies on electrospun nanofibers used in biomedical applications, where sizes in the range of 2–5 cm in length and 1 cm in width are frequently employed​. Using comparable specimen sizes ensures consistency with previously established methodologies in the field^61^. Moreover, A test rate of 8 mm/min is optimal for tensile testing of soft, fibrous materials like electrospun nanofibers^62^; this rate provides a balance between capturing accurate mechanical behavior and avoiding artifacts caused by rapid testing, such as premature failure due to high strain rates. In wound dressing studies involving electrospun PCL/PVP and other polymeric fibers, similar testing rates (e.g., 5–10 mm/min) have been reported, confirming that your choice aligns with the standard practices in this field^5^​. The stress-strain graph shows the mechanical behavior of the films subjected to stress from which the elastic modulus, tensile strength, and elongation at the break of the nanofibers are obtained. Due to the mechanical behavior of the nanofibers, it can be seen that there is an increase in the mechanical properties of *PCL/PVP* with *ChAgG* loads concerning PCL/PVP. However, it is observed that in the case of *PCL/PVP-ChAgG* loads at 10% only a decrease in the elastic modulus is appreciated. It should be noted that the best mechanical behavior of the nanofibers is obtained from *PCL/PVP-ChAgG* 5% with values ​​for the elastic modulus of 4.785 ± 0.430 MPa, tensile strength 1.706 ± 0.146, and elongation at break 126.483 ± 9.828% compared to *PCL/PVP* that has elastic modulus values ​​of 3.503 ± 0.512 MPa, tensile strength 0.735 ± 0.062 MPa and elongation at break 49.745 ± 18.471%.

As reported in the literature^63^, *PCL* and *PCL/Ag* electrospun nanofibers fabricated three samples with different concentrations of Ag (0.05, 0.25, 0.5, and 1 wt%), the sample with 0.05 wt% Ag presented a modulus of elasticity of 3.38 ± 0.74 MPa. Tensile strength of 1.72 ± 0.17 MPa and an elongation at break of 328 ± 12%, the 0.25 wt% Ag 4.28 ± 1.28 MPa, 2.78 ± 0.18%, and 427 ± 16 MPa respectively, but when reaching the sample of more excellent wt% Ag, these properties above decreased, presenting a modulus of elasticity of 2.97 ± 0.59 MPa, a tensile strength of 1.23 ± 0.08 MPa and an elongation at break of 251 ± 11%. When comparing the membrane (*PCL/Ag*) with *PCL*, the behavior of these results is similar to that of our fibers since the increase in the elastic modulus continues linearly until the sample with 0.25 wt% of the membranes containing Ag nanoparticles; however, increasing the content of silver nanoparticles caused a decrease in tensile strength and elongation at break. So, we can conclude that at a certain point, if *AgNPs* continue to be incorporated, the modulus of elasticity of the *PCL/PVP* membrane will decrease significantly; this is because the nanoparticles have high surface energy and are easy to agglomerate, which leads to their poor dispersion in the polymeric matrix.

On the other hand, it has been reported in the literature^64^, about fabricated and compared poly-caprolactone (*PCL*) and poly-caprolactone/graphene oxide (GO) fibrous membranes, where *PCL scaffolds*showed a tensile strength of 3.2 MPa, while those containing GO showed a higher one, up to 4 MPa and an elongation at break of approximately 100% and 120% respectively. The findings suggest that the inclusion of GO can enhance the scaffold’s structure, likely by enhancing interactions between the functional groups on the scaffold’s surface and the PCL matrix. Consequently, the unique mechanical properties of GO nanoparticles improve the stress-strain behavior of scaffolds, making them promising for wound healing applications. Similarly, our fibers exhibit comparable behavior when functionalized, indicating their potential for use in tissue regeneration. However, the studies conducted by Fahimirad and colleagues^65^, about *PCL* nanofibers incorporated with chitosan that presented a tensile strength with non-significant differences between both samples, of 7 MPa and an elongation at break of 35%, highlight the need for further enhancement. These results ​​obtained from the manufactured nanofibers demonstrate suitable mechanical tensile properties for application as a wound dressing but also stress the urgency for further improvement and innovation in this field.

In the other reports^66^, we conducted a manufacturing study comparing *PCL* and *PCL/PVP* fibers where *PCL/PVP* nanofibers showed a decrease in tensile strength compared to *PCL* nanofibers.

Finally^67^, proposals for a percolation-based model to analyze the conductivity of nanofiber composites were presented, and it was observed that material characteristics, such as the elastic moduli of nanoparticle-based composites, exhibit significant variations based on composition. Accordingly, sudden increments in modulus or conductivity are frequently interpreted within the context of percolation theory; this theory denotes the volume fraction at which particles form an interconnected network for the first time, elucidating the enhancement observed in the results demonstrated by the *PCL/PVP-ChAgG*5% membranes. However, a separate investigation^68^ carried out says that the characterization of the dispersion state of NPs in polymeric matrices is complicated at high concentrations, in addition to the fact that the polydispersity of NPs induces a slight decrease in the percolation threshold, which explains the behavior of *PCL/PVP-ChAgG*10% nanofibers; this is verified in the literature^69^, where they explain that the incorporation of the volume fraction of the nanoparticles in the polymeric matrix has a certain threshold level, and above that level, an aggregation is created in polymeric nanocomposites, which causes a reduction in the properties of the polymer.

On the other hand, In our previous work^5^. A dissolution test was conducted on the electrospun *PCL*,* PVP*, and *PCL/PVP* fibrous mats to assess how *PVP*dissolves within the PCL fibers. The procedure followed the methods described by Li X et al^70^. and Celebioglu, A., and Uyar, T^71^. For the test, 10 mg of each sample was placed in a glass Petri dish lined with lint-free absorbent paper soaked in 0.9% physiological solution (PISA) at room temperature and left for seven days. The dissolution of *PVP* in the *PCL* fibers was recorded using a Canon PC1304 semi-professional video camera mounted on a device that minimized shadows in the images. Each experiment was performed in triplicate. As expected, the *PCL* and *PCL/PVP* fibers showed no visible macroscopic degradation after seven days of observation.

In contrast, the *PVP* fibers dissolved almost immediately, within 14 s of being placed in the saturated physiological solution. SEM images of the *PCL/PVP* fibers, taken after seven days, showed holes throughout the *PCL* fibers, indicating the dissolution of *PVP*in the polymeric scaffolds^5^. In that previous study, the gradual degradation of *PCL* fibers was a beneficial characteristic for the dressings proposed. Meanwhile, PVP is recognized as a hydrogel that can absorb large amounts of water. Furthermore, *PVP*is intended to give the dressings a soft, non-adherent surface to prevent sticking to the wound^5^.

####  Antibacterial test (PCL/PVP and PCL/PVP-ChAgG fibers)

The concentrations of *ChAgG*(1%, 5%, and 10%) were likely chosen to investigate the balance between antimicrobial efficacy and biocompatibility in the functionalized fibers. The 1% concentration was selected to assess whether minimal amounts of antimicrobial components like silver nanocrystals, chitosan, and graphene oxide could still provide sufficient antibacterial action without inducing cytotoxicity. The 5% concentration, being intermediate, was likely chosen to achieve an optimal balance between antimicrobial properties and cell compatibility, based on previous studies^5,9^ suggesting that moderate amounts of silver and other agents offer strong antibacterial effects with manageable toxicity. The 10% concentration was tested to evaluate whether increasing the composite’s content could enhance antibacterial action further, while also monitoring for potential cytotoxic effects, which are more likely at higher concentrations. On the other hand, electrospun fibers cannot be formed after at higher concentration of 10% of the *ChAgG* nanocoposite.

**Figure 4:**
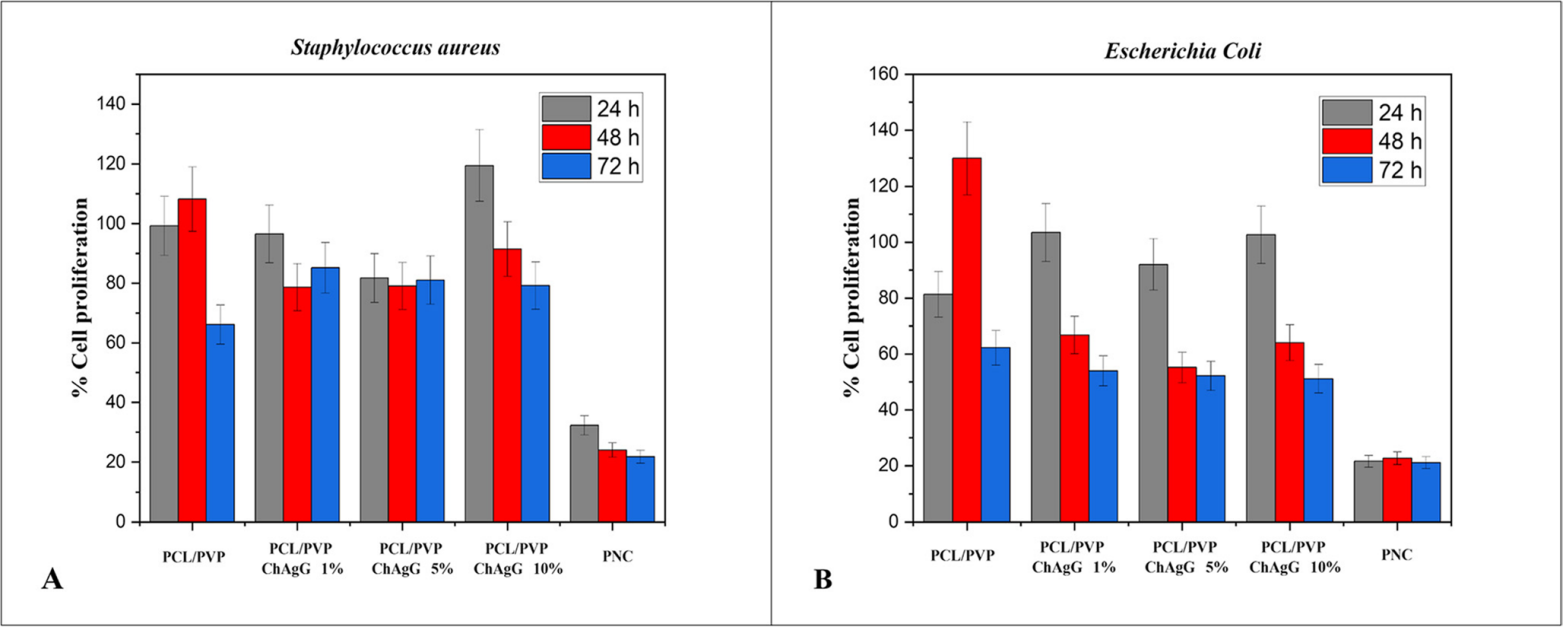
**(A)** Antibacterial study of *PCL/PVP* and *PCL/PVP-ChAgG* fibers against *Staphylococcus aureus* after 24, 48 and 72 h of exposition. Studies were made by triplicate. **(B)** Antibacterial study of *PCL/PVP* and *PCL/PVP-ChAgG* fibers against *Escherichia coli* after 24, 48 and 72 h of exposition. Studies were made by triplicate.

Gram-negative *Escherichia coli* and Gram-positive *Staphylococcus aureus* bacteria were chosen to assess the antibacterial efficacy of *PCL/PVP-ChAgG* fibers. Findings indicated that most of the produced fibers led to a reduction in bacterial population compared to the control (normal growth) when exposed to fibers loaded with the antibacterial compound (*ChAgG*) (*PCL/PVP-ChAgG*). However, variance analysis (ANOVA *P* > 0.05) revealed no significant difference in bacterial population growth between the functionalized and non-functionalized samples. Regarding samples exposed to *Staphylococcus aureus*, a notable decrease was observed after 48 h of exposure, particularly in fibers functionalized with 1% and 5% of the *ChAgG* compound (*PCL/PVP-ChAgG* 1% and *PCL/PVP-ChAgG* 5%, respectively) (Fig. 4). Conversely, *PCL/PVP-ChAgG* 10% membranes exhibited a significant increase in bacterial cell replication at 24 h, surpassing the control by 20%. However, after 48 and 72 h, the antibacterial activity of *PCL/PVP-ChAgG* 10% fibers led to a decrease of 10% and 20%, respectively. Notably, unfunctionalized *PCL/PVP* fibers did not significantly alter bacterial growth within the initial 48 h, but a 34% decrease was observed after 72 h. These findings suggest that functionalization of *PCL/PVP-ChAgG* fibers is generally less effective against *Staphylococcus aureus.*

On the contrary, when evaluating the *PCL/PVP* and *PCL/PVP-ChAgG* samples against *Escherichia coli* bacteria, a more pronounced antibacterial effect was observed than the study with *Staphylococcus aureus*. The *PCL/PVP* fibers exhibited a 30% increase in bacterial population at 48 h, but a decrease of 20% at 24 h and nearly 40% at 72 h of incubation was noted (Fig. 4B). In contrast, the functionalized fibers (*PCL/PVP-ChAgG*) showed a 3% increase in bacterial cell replication at 24 h. However, a gradual reduction in bacterial population of 34% at 48 h and 47% at 72 h was observed in the *PCL/PVP-ChAgG* 1% membranes. Similarly, in the *PCL/PVP-ChAgG* 10% membranes, there was a decrease of 36% at 48 h and 49% at 72 h, while in the *PCL/PVP-ChAgG* 5% fibers, a reduction of 45% at 48 h was observed. However, there was no significant difference in bacterial decrease at 72 h compared to 48 h.

The bacterial strains selected for this study could cause significant damage to the skin. For instance, *Staphylococcus aureus*is known for its association with skin and soft tissue infections in outpatient care^72^. At the same time, *Escherichia coli*can be found in surgical and traumatic wounds, foot ulcers, and decubitus ulcers^5^. These bacterial strains are commonly studied models and are often used for comparison due to their representation of different cell wall types—*E. coli* being Gram-negative and *S. aureus* Gram-positive.

The results revealed that the *PCL/PVP* and *PCL/PVP-ChAgG* fibers performed better against *Escherichia coli*. However, these membranes were less effective against S. aureus. One contributing factor to this discrepancy may be the multiple drug resistance phenotype of *S. aureus*, which makes it particularly challenging to treat^73^. The relatively low percentage of the antibacterial compound used in the membranes contributed to this outcome. Nonetheless, Chitosan-functionalized membranes demonstrated higher efficacy against *S. aureus* than *E. coli*, highlighting the differences in their cell wall structures. Previous studies have also shown that incorporating chitosan with silver ions (Ag) or nanoparticles results in more significant antibacterial activity than using each component individually^74,75^.

Although PCL and PVP polymers themselves lack intrinsic antibacterial properties, they have been found to influence bacterial growth 5. While the membranes formed from the PCL/PVP mixture showed slight antibacterial activity against *E. coli* and *S. aureus*, the effects were not as pronounced.

It has been documented that the antibacterial properties of graphene-based surfaces can be enhanced by incorporating silver, zinc, and iron nanoparticles^76^. In this context, our *PCL/PVP-ChAgG* membranes exhibited enhanced effectiveness against *E. coli*, aligning with results reported in similar studies where the combination of graphene with chitosan demonstrated more potent inhibition effects dependent on the concentration of these components^77^.

Again, the effectiveness of the functionalized fibers against *Escherichia coli* (Gram-negative) compared to *Staphylococcus aureus*(Gram-positive) can be explained by the structural differences in their cell walls, which play a significant role in their susceptibility to antibacterial agents^78^. Gram-negative bacteria like *E. coli*have a relatively thin peptidoglycan layer surrounded by an outer membrane composed of lipopolysaccharides (LPS); this outer membrane, while providing some protection, is more permeable to antibacterial agents such as silver ions and reactive oxygen species (ROS). Consequently, Gram-negative bacteria are more vulnerable to mechanisms that disrupt cell membrane integrity and interfere with essential metabolic processes^79^.

In contrast, Gram-positive bacteria like *S. aureus*possess a much thicker peptidoglycan layer, providing them with greater mechanical strength and a more substantial barrier to external threats; this thicker cell wall, which lacks an outer membrane, makes it more difficult for antibacterial agents to penetrate and reach vital cellular targets^80^. As a result, *S. aureus* displays more resistance to the antibacterial effects of the functionalized fibers compared to *E. coli*^81^.

The functionalized fibers contain silver nanocrystals (AgNCs), chitosan, and graphene oxide (GO), each contributing to a multi-modal antibacterial effect. Silver ions (Ag⁺), released from the nanocrystals, bind to bacterial cell membranes, causing structural damage and altering permeability. Once inside the cell, silver ions inhibit critical processes such as DNA replication, protein synthesis, and enzyme activity, leading to bacterial cell death. The thinner cell wall and outer membrane of *E. coli* allow silver ions to penetrate more easily, making this bacterium more susceptible to their effects. On the other hand, *S. aureus*’s thicker peptidoglycan layer likely impedes the penetration of silver ions, reducing their antibacterial efficacy^4,82^.

Chitosan, another component of the fibers, interacts with the negatively charged bacterial membrane, altering its permeability and leading to leakage of intracellular contents; this electrostatic interaction is more effective against Gram-negative bacteria like *E. coli*, which have a less robust membrane structure. In contrast, the thicker peptidoglycan layer of *S. aureus*offers additional protection, reducing the extent of chitosan-induced membrane disruption^83^.

Graphene oxide (GO) contributes both mechanical and chemical antibacterial effects. Its sharp edges can physically disrupt bacterial membranes, while it also generates reactive oxygen species (ROS) that cause oxidative stress within bacterial cells. Gram-negative bacteria, with their thin peptidoglycan layer, are more vulnerable to these mechanical and oxidative disruptions. The thick peptidoglycan layer of *S. aureus*acts as a buffer, protecting the bacterial cells from the full impact of graphene oxide^84^.

The combination of silver nanocrystals, chitosan, and graphene oxide creates a powerful synergistic effect against *E. coli*. Silver ions attack the bacterial DNA and proteins, chitosan disrupts the membrane, and graphene oxide induces mechanical damage and oxidative stress. Together, these actions lead to rapid and significant bacterial cell death in *E. coli*. However, *S. aureus*’s thick peptidoglycan layer slows the penetration of these agents, reducing their overall antibacterial effect. Additionally, *S. aureus*may possess greater resistance to oxidative stress caused by graphene oxide^85^.

The observed differences in antibacterial activity between *E. coli* and *S. aureus* reflect the unique structural characteristics of Gram-negative and Gram-positive bacteria. The thinner, more permeable cell wall of *E. coli* allows antibacterial agents to act more effectively, while the thick peptidoglycan layer of *S. aureus*provides greater resistance. Understanding these differences highlights the importance of tailoring antibacterial treatments to target specific bacterial vulnerabilities for more effective results^78^.

####  MTT cell viability test (PCL/PVP and PCL/PVP-ChAgG fibers)

**Figure 5:**
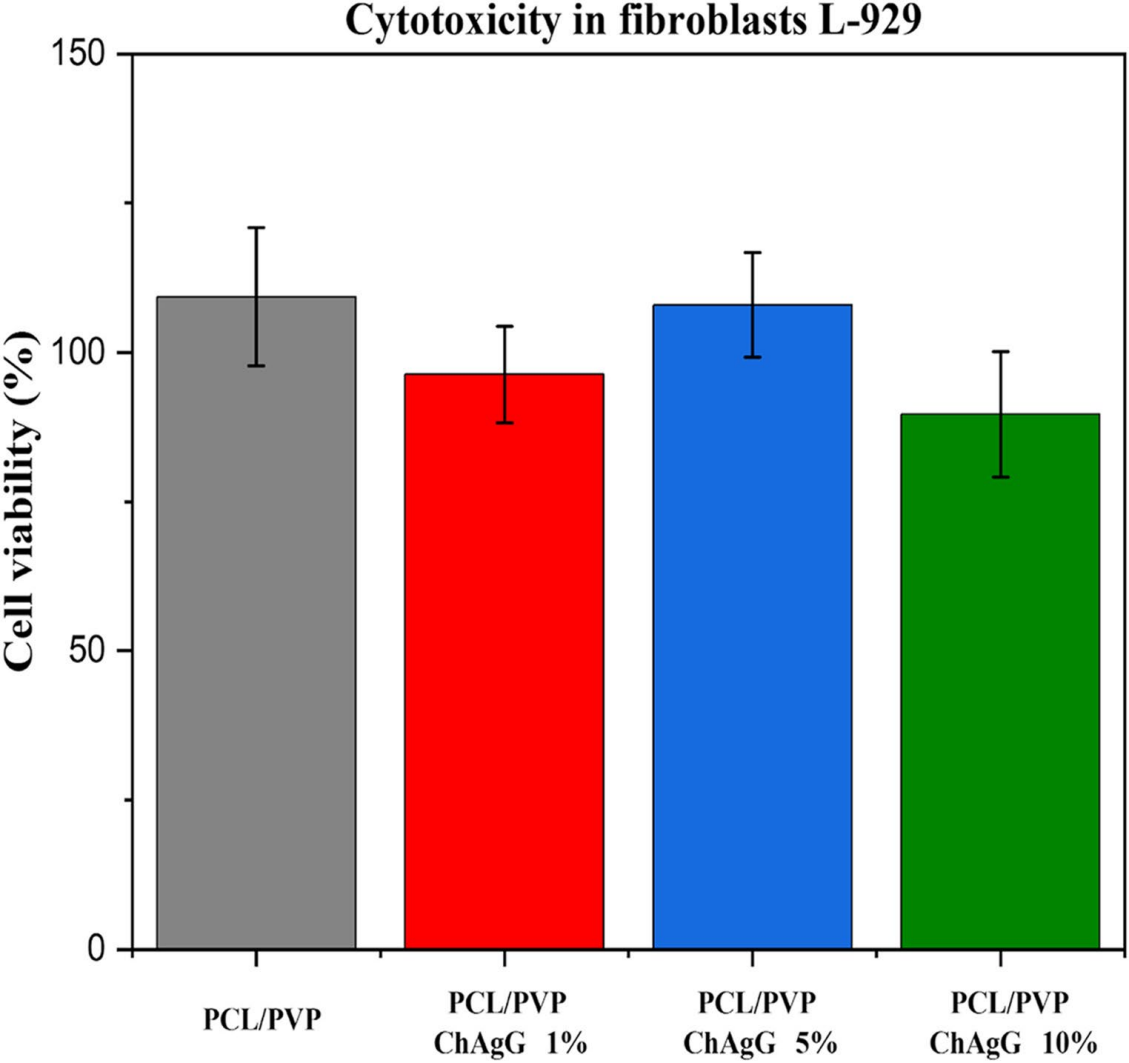
MTT cell viability test of *PCL/PVP* and *PCL/PVP-ChAgG* fibers after 24 h of exposition. Studies were made by triplicate.

A colorimetric assay using the MTT technique was conducted for the cytotoxicity assessment to measure cellular metabolic activity with L-929 mouse fibroblasts. After 24 h of incubation, no notable difference in cell proliferation was observed compared to the control fibers (*PCL/PVP*) (Fig. 5).

The proliferation of L-929 cultures indicated that non-functionalized fibers (*PCL/PVP*) promoted proliferation by 109 ± 11%. Conversely, fibers loaded with the antibacterial compound (*ChAgG*), specifically *PCL/PVP-ChAgG* 1%, showed a 96 ± 3% proliferation. Similarly, membranes containing *PCL/PVP-ChAgG* 5% exhibited a proliferation of 108 ± 11%, which closely resembled the result of non-functionalized fibers (*PCL/PVP*). Finally, *PCL/PVP-ChAgG* 10% fibers showed a 90 ± 9% proliferation. These findings suggest that the *PCL/PVP* complex and the fibrous structure did not disrupt the expected growth of L929 fibroblasts. Additionally, the addition of the *ChAgG* compound did not induce significant alterations, with only slight toxicity observed at 1% and 10%, while an increase in cell growth was evident at 5%. Nevertheless, these minor changes in cell behavior do not indicate a substantial impact.

The ANOVA revealed that after 24 h, there were no significant alterations in the normal growth of L929 fibroblasts when exposed to either *PCL/PVP* or *PCL/PVP-ChAgG* fibers. This suggests that both types of fibers are non-toxic to the human body, a reassuring finding for potential applications.

L929 cells were selected for this study due to their well-established culture protocols, reproducibility, and ease of proliferation; this cell line is commonly employed for initial cytotoxicity assessments of various biomaterials because of its robust growth and ability to adhere to most biomaterial surfaces^86^.

Phenol was chosen as the positive control in this study due to its well-established cytotoxic and antimicrobial properties. It is a strong disinfectant and antiseptic that effectively disrupts bacterial cell membranes and denatures proteins, leading to cell death. Phenol’s potent antimicrobial action and toxicity make it a reliable reference point when evaluating the safety and efficacy of the *PCL/PVP-ChAgG*functionalized fibers. By using phenol, our study set a high standard for antibacterial effectiveness while also ensuring that any cytotoxicity observed in the test fibers does not exceed the known harmful effects of phenol; this comparison allows for a better understanding of how the experimental fibers balance antimicrobial activity with biocompatibility^87^.

Other potential positive controls could also be used depending on the specific objectives of the study. For example, silver-based antiseptics like silver sulfadiazine would provide a relevant comparison given that the functionalized fibers contain silver nanocrystals. Silver sulfadiazine is commonly used in wound care for its broad-spectrum antimicrobial properties, and using it as a control would allow for a direct comparison of the fibers’ efficacy with a clinically utilized silver-based treatment^88^.

Chlorhexidine is another widely used antiseptic, particularly in wound care and surgical settings. It has broad-spectrum antimicrobial activity with relatively lower cytotoxicity compared to phenol. Chlorhexidine would be a useful control to compare the antimicrobial effectiveness of the fibers without the high toxicity levels associated with phenol; this could provide valuable insights into the biocompatibility of the *PCL/PVP-ChAgG*fibers^89^.

Specific antibiotics, such as penicillin or vancomycin, could also serve as positive controls, especially when targeting bacterial strains like *Staphylococcus aureus* or *Escherichia coli*. Using antibiotics would offer a comparison of the antimicrobial properties of the fibers relative to conventional treatments and could provide additional context on how the fibers perform against resistant bacterial strains^90^.

Finally, hydrogen peroxide could be used as a positive control because it is a common wound cleaning agent. It works by generating reactive oxygen species that kill bacteria; this would allow for a comparison between the oxidative stress-inducing capabilities of hydrogen peroxide and the graphene oxide in the fibers, which also produces reactive oxygen species as part of its antimicrobial mechanism^91^. Each of these controls would provide valuable context for evaluating the antibacterial and cytotoxic properties of the *PCL/PVP-ChAgG* fibers, depending on the focus of the research and the bacterial strains under investigation.

The MTT assay, utilized to gauge cellular metabolic activity, relies on detecting a color change^86^; this process entails the conversion of the yellow tetrazolium salt, MTT (3-(4,5-dimethylthiazol-2-yl)-2,5-diphenyltetrazolium bromide), into a purple formazan product^92^ examined electrospun membranes incorporating quercetin into a matrix of poly (caprolactone) (PCL) and poly (vinyl pyrrolidone) (PVP) for potential health applications. These membranes were produced via uniaxial electrospinning, with a quercetin concentration of 15% by weight. Morphological analysis confirmed the fabrication of micrometer-sized fibers, measuring 1.58 μm for the PCL/PVP composite and 2.34 μm for the quercetin-loaded membrane. The biological activity of the released quercetin was assessed using two human cell lines, indicating that the quercetin-loaded membranes could reduce cell viability under direct contact and exposure conditions. These initial findings underscore the potential utility of electrospun PCL-PVP membranes loaded with quercetin in healthcare applications, promising significant advancements in the field.

Nanofibers are extensively utilized in biomedical fields to create intricate 3D-fibrous structures. Poly (caprolactone) (PCL) is a semi-crystalline polymer widely employed in surgical sutures, drug delivery systems, and tissue regeneration scaffolds. PCL offers advantages such as biodegradability, ease of processing, and favorable mechanical properties. Its degradation involves a two-stage process: initial non-enzymatic hydrolysis of the aliphatic ester group through surface bulk degradation pathways, followed by phagocytosis by macrophages and giant cells, leading to intracellular degradation and the production of hydrolysis intermediates. These intermediates are eventually excreted from the body, resulting in the gradual and sustained degradation of PCL over an extended period, spanning several months to years^93^.

To be effective, wound dressings should possess antimicrobial properties to shield the wound from contamination and minimize the risk of secondary infections. This is a crucial aspect of wound care that has significant implications for patient recovery and healthcare costs. Different methods have been investigated to achieve this goal, including incorporating antibiotics and utilizing materials with inherent antimicrobial properties^94^.

The mild toxicity observed at 1% and 10% *ChAgG* concentrations could have several biological explanations related to the interaction of these components with cells. Each component of the *ChAgG*composite has distinct properties that may contribute to cytotoxicity, particularly at higher concentrations, and these effects could be magnified when combined^4^.

One potential explanation is the over-release of silver ions (Ag⁺) at these concentrations. Silver ions are known for their strong antibacterial properties, but they can also cause oxidative stress in mammalian cells by generating reactive oxygen species (ROS). At lower concentrations (such as 1%); this oxidative stress could begin to disrupt cellular processes without causing immediate cell death, leading to mild toxicity. At higher concentrations (10%), silver ion accumulation could cause more significant cellular damage, leading to decreased cell viability. To further investigate this hypothesis, experiments measuring ROS production in cells exposed to different *ChAgG*concentrations could be conducted. Additionally, studying the dose-dependent release of silver ions would provide insight into how silver concentration correlates with cytotoxicity^95,96^.

Another explanation could be membrane disruption due to chitosan and graphene oxide. Chitosan is positively charged and interacts with negatively charged cell membranes, potentially leading to increased membrane permeability or even membrane disruption at higher concentrations; this disruption can interfere with cellular homeostasis and result in cytotoxic effects. Similarly, graphene oxide’s sharp edges can physically damage cell membranes, and in higher concentrations; this damage could be more pronounced. Investigating these mechanisms could involve microscopy techniques such as scanning electron microscopy (SEM) or transmission electron microscopy (TEM) to observe any morphological changes in cell membranes after exposure to *ChAgG*. Additionally, membrane integrity assays could help quantify the extent of membrane disruption^97^.

At higher concentrations, graphene oxide may also generate higher oxidative stress, which could exacerbate the observed toxicity. Graphene oxide has been reported to cause mitochondrial dysfunction and oxidative stress through ROS production, which can damage proteins, lipids, and DNA within cells. To investigate this, experiments could be designed to assess mitochondrial health and oxidative stress markers in cells treated with varying *ChAgG*concentrations^98^.

Lastly, agglomeration of nanoparticles at higher concentrations could play a role in toxicity. As the concentration of nanoparticles (such as silver and graphene oxide) increases, their tendency to aggregate may also increase, reducing their dispersion within the polymer matrix; this could lead to localized areas of high nanoparticle concentration, resulting in cytotoxic hotspots. Future studies could explore the dispersion state of the nanoparticles within the fibers using techniques like dynamic light scattering (DLS) or X-ray diffraction (XRD); this would help confirm if agglomeration is contributing to the observed toxicity^99^.

To further investigate these mechanisms, a combination of cytotoxicity assays, oxidative stress measurements, and membrane integrity studies could be employed. By conducting these analyses, researchers could better understand the concentration-dependent toxicity of *ChAgG*fibers and optimize the balance between antimicrobial efficacy and biocompatibility^100^.

Future studies should focus on the long-term effects of these functionalized fibers on cell viability and proliferation, especially in the context of chronic wound healing. Extended cell culture studies using fibroblasts, keratinocytes, or other relevant cell lines could assess whether prolonged exposure to the fibers promotes or inhibits cellular growth and differentiation. Evaluating the fibers over extended periods (e.g., weeks to months) would help determine their biocompatibility, cytotoxicity, and potential for enhancing tissue regeneration. Additionally, exploring the release kinetics of antibacterial agents such as silver ions and their cumulative impact on cell behavior would provide valuable insights into how the fibers interact with wound environments over time^101^.

In vivo studies should also be conducted to evaluate the biocompatibility of these fibers in animal models; this would involve testing the fibers on wound sites in animals to monitor tissue integration, inflammatory responses, and overall healing outcomes. Investigating factors such as wound closure rates, scar formation, and potential adverse effects would help confirm the fibers’ suitability for real-world wound dressing applications. Moreover, examining how the fibers degrade in vivo and their impact on surrounding tissues would be crucial for ensuring their safe and effective use in clinical settings. These studies would provide essential data on the fibers’ long-term performance, safety, and potential for translation into medical use^102^.

## Conclusions

Bioactive wound dressings are pivotal in managing superficial skin and burn injuries, particularly those that generate excessive exudate; this study has addressed the challenges of microbial contamination and secure adhesion of dressings to the affected areas, which is essential for promoting skin healing. We developed an electrospun wound dressing enhanced with a synthesized bioactive compound, *ChAgG*, composed of chitosan, silver nanocrystals, and graphene oxide. These components confer bioactive properties critical for effective wound management. Comprehensive characterizations, including FTIR, XPS, Raman, and TEM analyses, validated the successful integration of these bioactive agents, confirming the production of silver crystals and oxidation of graphene, essential for the nanocomposite’s functionality. Furthermore, the functionalization of *PCL/PVP* electrospun nanofibers with the *ChAgG* compound was evidenced through FTIR, TGA-DSC, and SEM-EDX techniques. The dressing’s structural integrity and interconnected porosity were elucidated via SEM and X-ray tomography, demonstrating a three-dimensional configuration conducive to tissue integration and healing. Mechanical testing indicated optimal properties with a 5% w/v *ChAgG* concentration, enhancing both the fibers’ antimicrobial efficacy and mechanical strength, with superior performance noted against *Escherichia coli* compared to *Staphylococcus aureus.* Future directions will focus on expanded biological assessments including exposure of the *ChAgG* dressing to fibroblast and blood cells, implantation in murine models, evaluation of immunological responses, and further validation of its antimicrobial capabilities against targeted pathogens; this progression aims to bridge the gap identified in foundational research, enhancing the translation of our bioactive wound dressing from laboratory research to clinical application, thereby improving outcomes in wound management globally. Hence, the primary objective of developing *PCL/PVP*-*ChAgG* functionalized fibers as an advanced wound dressing solution was to enhance antimicrobial efficacy while maintaining biocompatibility and mechanical strength. The conclusions show that the fibers succeeded in providing effective antibacterial action, particularly against *Escherichia coli*, and demonstrated favorable mechanical properties, thereby aligning with the goal of creating durable and effective wound dressings. However, the observed mild cytotoxicity at higher *ChAgG* concentrations and varying efficacy against different bacterial strains point to areas for further refinement, as outlined in the study’s limitations.

Among the limitations of the study, are the absence of long-term cytotoxicity data and the need for more comprehensive in vivo studies to fully assess the biocompatibility, degradation rates, and overall effectiveness of the *PCL/PVP-ChAgG* functionalized fibers in wound healing. Additionally, the variation in antibacterial performance between *Escherichia coli* and *Staphylococcus aureus* suggests that the material may not be equally effective against all bacterial strains, requiring further investigation into its broad-spectrum antibacterial efficacy. Another limitation is the lack of comparative studies with other clinically used wound dressings, which would provide a clearer picture of how this material performs relative to current standards. Despite these limitations; this new material shows significant potential in wound dressing applications due to its multifunctional properties. The integration of chitosan, silver nanocrystals, and graphene oxide provides a powerful combination of antimicrobial efficacy, biocompatibility, and mechanical strength, which could make it highly effective in treating wounds prone to infection, particularly chronic and burn wounds. Compared to existing wound dressings, the *PCL/PVP-ChAgG* functionalized fibers offer sustained antimicrobial action, controlled degradation, and the ability to promote tissue regeneration, positioning them as a promising alternative to traditional options such as gauze, hydrogels, or silver-based dressings. Further research and development could enhance its applicability, making it a strong candidate for next-generation wound care solutions.

The clinical translation of *PCL/PVP-ChAgG* enhanced wound dressings will face regulatory hurdles, primarily from agencies like the FDA or EMA, requiring extensive preclinical and clinical data on biocompatibility, safety, and long-term efficacy. The presence of nanomaterials, such as silver nanocrystals and graphene oxide, may invite stricter scrutiny due to concerns over toxicity, bioaccumulation, and environmental impact. Addressing these issues will involve thorough toxicity studies and establishing robust quality control for manufacturing consistency. Commercialization strategies could involve partnerships with medical device companies to leverage their regulatory experience and resources for scaling up production and conducting clinical trials. Securing intellectual property protection, obtaining funding, and conducting cost-effectiveness assessments will be crucial. Demonstrating the product’s ability to reduce infection rates, enhance healing, and minimize dressing changes could make it an attractive alternative in the wound care market.

## Supplementary Information

Supplementary Material 1.

## Data Availability

The data used to support the findings of this study are included within the article and it are available from the corresponding author upon request.

## References

[1] Forbinake, N. A. et al. Mortality analysis of burns in a developing country: a CAMEROONIAN experience. *BMC Public. Health*. **20**, 1269 (2020). 32819340 10.1186/s12889-020-09372-3 PMC7441696

[2] Roshangar, L., Soleimani Rad, J., Kheirjou, R. & Reza Ranjkesh, M. Ferdowsi Khosroshahi, A. Skin Burns: review of Molecular mechanisms and therapeutic approaches. *Wounds: Compendium Clin. Res. Pract. ***31**, 308–315 (2019). 31730513

[3] Nguyen, H. M., Le, N., Nguyen, T. T., Le, A. T. T., Pham, T. T. & H. N. & Biomedical materials for wound dressing: recent advances and applications. *RSC Adv. ***13**, 5509–5528 (2023). 36793301 10.1039/d2ra07673j PMC9924226

[4] Estevez Martínez, Y., Vázquez Mora, R., Méndez Ramírez, Y.I., Chavira Martínez, E., Huirache Acuña, R., Díaz de León Hernández, J.N. & Villarreal Gómez, L.J. Antibacterial nanocomposite of chitosan/silver nanocrystals/graphene oxide (ChAgG) development for its potential use in bioactive wound dressings. *Sci. Rep.* **13** (1), 10234 (2023). 10.1038/s41598-023-29015-y PMC10290094 37353546

[5] Álvarez-Suárez, A. S. et al. Electrospun Fibers and sorbents as a possible basis for effective composite wound dressings. *Micromachines*. **11**, 441 (2020). 32331467 10.3390/mi11040441 PMC7231366

[6] Huo, S. et al. Graphene oxide with acid-activated bacterial membrane anchoring for improving synergistic antibacterial performances. *Appl. Surf. Sci. ***551**, 149444 (2021).

[7] Bruna, T., Maldonado-Bravo, F., Jara, P. & Caro, N. Silver nanoparticles and their antibacterial applications. *IJMS*. **22**, 7202 (2021). 34281254 10.3390/ijms22137202 PMC8268496

[8] Rajinikanth, B., Rajkumar, S., K, D. S. R., Vijayaragavan, V. & K. & Chitosan-based Biomaterial in Wound Healing: a review. *Cureus*. 10.7759/cureus.55193 (2024). 10.7759/cureus.55193 PMC10983058 38562272

[9] Mendoza Villicana, A. et al. Evaluation of strategies to incorporate silver nanoparticles into electrospun microfibers for the preparation of wound dressings and their antimicrobial activity. *Polymer-Plastics Technol. Mater. ***62**, 1029–1056 (2023).

[10] Brumberg, V., Astrelina, T., Malivanova, T. & Samoilov, A. *Mod. Wound Dressings: Hydrogel Dressings Biomedicines. ***9**, 1235 (2021). 10.3390/biomedicines9091235 PMC8472341 34572421

[11] Hussain, S. & Maktedar, S. S. Structural, functional and mechanical performance of advanced graphene-based composite hydrogels. *Results Chem. ***6**, 101029 (2023).

[12] Sood, A., Granick, M. S. & Tomaselli, N. L. Wound dressings and comparative Effectiveness Data. *Adv. Wound Care*. **3**, 511–529 (2014). 10.1089/wound.2012.0401 PMC4121107 25126472

[13] Rather, A. H., Khan, R. S., Rafiq, M., Tripathi, R. M. & Sheikh, F. A. Polyurethane nanofibers incorporated magnesium hydroxide followed by hydrothermal treatment using chitosan and silver nanoparticles to improve the biological properties. *J. Appl. Polym. Sci. ***141**, e55735 (2024).

[14] Estévez Martínez, Y., Vázquez Mora, R. & Chavira Martínez, E. *Método para Sintetizar Nanocristales de Plata a Partir de Plantas Amarilidaceas (Amaryllidaceae)* (2020).

[15] Abe, K., Hori, Y. & Myoda, T. Volatile compounds of fresh and processed garlic (review). *Exp. Ther. Med. *10.3892/etm.2019.8394 (2019). 10.3892/etm.2019.8394 PMC6966211 32010343

[16] Bouqellah, N. A., Mohamed, M. M. & Ibrahim, Y. Synthesis of eco-friendly silver nanoparticles using Allium sp. and their antimicrobial potential on selected vaginal bacteria. *Saudi J. Biol. Sci. ***26**, 1789–1794 (2019). 31762659 10.1016/j.sjbs.2018.04.001 PMC6864148

[17] Ul-Islam, M. et al. Chitosan-based nanostructured biomaterials: synthesis, properties, and biomedical applications. *Adv. Industrial Eng. Polym. Res. ***7**, 79–99 (2024).

[18] Badoni, A. & Prakash, J. Noble metal nanoparticles and graphene oxide based hybrid nanostructures for antibacterial applications: recent advances, synergistic antibacterial activities, and mechanistic approaches. *Micro Nano Eng. ***22**, 100239 (2024).

[19] Jaworski, S. et al. Graphene Oxide-based nanocomposites decorated with silver nanoparticles as an Antibacterial Agent. *Nanoscale Res. Lett. ***13**, 116 (2018). 29687296 10.1186/s11671-018-2533-2 PMC5913058

[20] Crist, B. V. A Review of XPS Data-Banks. *Surf. Interface Anal*. **1**, 1–52 (2007).

[21] Wang, L. et al. Combined TPD and XPS Study of Ligation and Decomposition of 1,6-Hexanedithiol on size-selected copper clusters supported on HOPG. *J. Phys. Chem. C*. **122**, 2173–2183 (2018).

[22] Isaacs, M. A. et al. Advanced XPS characterization: XPS-based multi-technique analyses for comprehensive understanding of functional materials. *Mater. Chem. Front. ***5**, 7931–7963 (2021).

[23] Estévez-Martínez, Y. et al. Click chemistry of multi-walled carbon nanotubes-g-1,3-diazido-2-isopropanol with alkyne groups. *Rev. Adv. Mater. Sci.* **52**, 18–28 (2017).

[24] Boronin, A. I., Koscheev, S. V. & Zhidomirov, G. M. XPS and UPS study of oxygen states on silver. *J. Electron Spectrosc. Relat. Phenom. ***96**, 43–51 (1998).

[25] Streletskiy, O. et al. Tailoring of the distribution of SERS-Active silver nanoparticles by Post-deposition Low-Energy Ion Beam Irradiation. *Materials*. **15**, 7721 (2022). 36363312 10.3390/ma15217721 PMC9659245

[26] Guo, F. et al. Characterization of organic matter of plants from lakes by thermal analysis in a N2 atmosphere. *Sci. Rep. ***6**, 22877 (2016). 26953147 10.1038/srep22877 PMC4782168

[27] Al-Gaashani, R., Najjar, A., Zakaria, Y., Mansour, S. & Atieh, M. A. XPS and structural studies of high quality graphene oxide and reduced graphene oxide prepared by different chemical oxidation methods. *Ceram. Int. ***45**, 14439–14448 (2019).

[28] Jiang, H., Srichuwong, S., Campbell, M. & Jane, J. Characterization of maize amylose-extender (ae) mutant starches. Part III: structures and properties of the Naegeli dextrins. *Carbohydr. Polym. ***81**, 885–891 (2010).

[29] Pal, N., Banerjee, S., Roy, P. & Pal, K. Cellulose nanocrystals–silver nanoparticles-reduced graphene oxide based hybrid PVA nanocomposites and its antimicrobial properties. *Int. J. Biol. Macromol. ***191**, 445–456 (2021). 34555401 10.1016/j.ijbiomac.2021.08.237

[30] Tanaka, K. & Iijima, S. *Carbon nanotubes and Graphene*. (Elsevier, 2014) 10.1016/C2011-0-07380-5.

[31] Wu, Z., Huang, X., Li, Y. C., Xiao, H. & Wang, X. Novel chitosan films with laponite immobilized Ag nanoparticles for active food packaging. *Carbohydr. Polym. ***199**, 210–218 (2018). 30143123 10.1016/j.carbpol.2018.07.030

[32] Kotsyubynsky, V. O. et al. Structural, morphological and electrical properties of graphene oxides obtained by hummers, Tour and modified methods: a comparative study. *Phys. Chem. Solid St*. **22**, 31–38 (2021).

[33] Zając, A., Hanuza, J., Wandas, M. & Dymińska, L. Determination of N-acetylation degree in chitosan using Raman spectroscopy. *Spectrochim. Acta Part A Mol. Biomol. Spectrosc.* **134**, 114–120 (2015). 10.1016/j.saa.2014.06.071 25011040

[34] Kang, Y., Kim, H. J., Lee, S. H. & Noh, H. Paper-Based Substrate for a Surface-Enhanced Raman Spectroscopy Biosensing Platform—A Silver/Chitosan Nanocomposite Approach. *Biosensors* **12**, 266 (2022). 35624567 10.3390/bios12050266 PMC9138243

[35] Feng, C. et al. Silver nanoparticle-decorated Chitosan Aerogels as three-dimensional porous surface-enhanced Raman Scattering substrates for Ultrasensitive Detection. *ACS Appl. Nano Mater. ***5**, 5398–5406 (2022).

[36] Akashi, L. et al. Interaction of Silver nanoparticles with Bilayer Graphene: a Raman Study. *Braz J. Phys. ***52**, 116 (2022).

[37] Bonilla, J., Atarés, L., Vargas, M. & Chiralt, A. Properties of wheat starch film-forming dispersions and films as affected by Chitosan addition. *J. Food Eng. ***114**, 303–312 (2013).

[38] Arroyo, G., Angulo, Y., Debut, A. & Cumbal, L. H. Synthesis and characterization of silver nanoparticles prepared with Carrasquilla Fruit Extract (Berberis hallii) and evaluation of its photocatalytic activity. *Catalysts*. **11**, 1195 (2021).

[39] Courty, A. Silver nanocrystals: Self-Organization and collective properties. *J. Phys. Chem. C*. **114**, 3719–3731 (2010).

[40] Asanithi, P., Chaiyakun, S. & Limsuwan, P. Growth of silver nanoparticles by DC Magnetron Sputtering. *J. Nanomaterials*. **2012**, 1–8 (2012).

[41] Murthy, H. C. A., Zeleke, D., Ravikumar, T., Anil Kumar, C. R., Nagaswarupa, H. P. & M. R. & Electrochemical properties of biogenic silver nanoparticles synthesized using Hagenia Abyssinica (Brace) JF. Gmel. Medicinal plant leaf extract. *Mater. Res. Express*. **7**, 055016 (2020).

[42] Ali, A. & Ahmed, S. A review on chitosan and its nanocomposites in drug delivery. *Int. J. Biol. Macromol. ***109**, 273–286 (2018). 29248555 10.1016/j.ijbiomac.2017.12.078

[43] Khan, M., Shameli, K., Sazili, A., Selamat, J. & Kumari, S. Rapid Green Synthesis and characterization of silver nanoparticles arbitrated by Curcumin in an Alkaline Medium. *Molecules*. **24**, 719 (2019). 30781541 10.3390/molecules24040719 PMC6412299

[44] Chung, I. M., Park, I., Seung-Hyun, K., Thiruvengadam, M. & Rajakumar, G. Plant-mediated synthesis of silver nanoparticles: their characteristic properties and therapeutic applications. *Nanoscale Res. Lett. ***11**, 40 (2016). 26821160 10.1186/s11671-016-1257-4 PMC4731379

[45] Kalwar, K. & Shan, D. Antimicrobial effect of silver nanoparticles (AgNPs) and their mechanism – a mini review. *Micro Nano Lett. ***13**, 277–280 (2018).

[46] Varsei, M., Tanha, N. R., Gorji, M. & Mazinani, S. Fabrication and optimization of PCL/PVP nanofibers with Lawsonia inermis for antibacterial wound dressings. *Polym. Polym. Compos. ***29**, S1403–S1413 (2021).

[47] Cao, Y. et al. Polycaprolactone/polyvinyl pyrrolidone nanofibers developed by solution blow spinning for encapsulation of chlorogenic acid. *Food Qual. Saf. ***6**, 1–10 (2022).

[48] Kosowska, K. et al. Gradient chitosan hydrogels modified with graphene derivatives and hydroxyapatite: physiochemical properties and initial cytocompatibility evaluation. *Int. J. Mol. Sci. ***21**, 1–21 (2020). 10.3390/ijms21144888 PMC7404139 32664452

[49] Mohammadzadeh Kakhki, R., Hedayat, S. & Mohammadzadeh, K. Novel, green and low cost synthesis of Ag nanoparticles with superior adsorption and solar based photocatalytic activity. *J. Mater. Sci. Mater. Electron. *10.1007/s10854-019-01203-5 (2019).

[50] Caro, C. A. et al. Preparation, spectroscopic, and electrochemical characterization of metal(II) complexes with Schiff base ligands derived from Chitosan: correlations of redox potentials with Hammett parameters. *J. Coord. Chem. ***67**, 4114–4124 (2014).

[51] Kosowska, K., Domalik-Pyzik, P., Krok-Borkowicz, M. & Chłopek, J. Polylactide/hydroxyapatite nonwovens incorporated into chitosan/graphene materials hydrogels to form novel hierarchical scaffolds. *Int. J. Mol. Sci. ***21 **(7), 23–30 (2020). 10.3390/ijms21072330 PMC7178071 32230916

[52] Li, X. M. et al. Fabrication of chitosan hydrochloride and carboxymethyl starch complex nanogels as potential delivery vehicles for curcumin. *Food Chem. ***293**, 197–203 (2019). 31151601 10.1016/j.foodchem.2019.04.096

[53] Li, X. M. et al. Chitosan hydrochloride/carboxymethyl starch complex nanogels as novel Pickering stabilizers: physical stability and rheological properties. *Food Hydrocoll. ***93**, 215–225 (2019).

[54] França, D. C., Bezerra, E. B., Morais, D. D. S., Araújo, E. M. & Wellen, R. M. R. *Effect of Hydrolytic Degradation on Mechanical Properties of PCL*. *Mater. Sci. Forum* vol. 869 345 (Trans Tech Publications Ltd, Switzerland, 2016).

[55] França, D. C., Bezerra, E. B., De Souza Morais, D. D., Araújo, E. M. & Wellen, R. M. R. Hydrolytic and thermal degradation of PCL and PCL/bentonite compounds. *Mater. Res. ***19**, 618–627 (2016).

[56] Chadha, R., Kapoor, V. K. & Kumar, A. Analytical techniques used to characterize drug-polyvinylpyrrolidone systems in solid and liquid states - an overview. *J. Sci. Ind. Res. ***65**, 459–469 (2006).

[57] D’Amelia, R. P., Gentile, S., Nirode, W. F. & Huang, L. Quantitative analysis of copolymers and blends of polyvinyl acetate (PVAc) using Fourier transform infrared spectroscopy (FTIR) and elemental analysis (EA). *World J. Chem. Educ. ***4**, 25–31 (2016).

[58] Jaganathan, S. K. & Mani, M. P. Electrospinning synthesis and assessment of physicochemical properties and biocompatibility of cobalt nitrate fibers for wound healing applications. *Anais Acad. Bras. Cienc. ***91**, e20180237 (2019). 10.1590/0001-3765201920180237 31365648

[59] Williams, C. III et al. Cerium(III) nitrate containing electrospun wound dressing for mitigating burn severity. *Polym. ***13 **(18), 31–74 (2021). 10.3390/polym13183174 PMC8470165 34578075

[60] Zhao, J. et al. Chitosan, N,N,N-trimethyl Chitosan (TMC) and 2-hydroxypropyltrimethyl ammonium chloride chitosan (HTCC): the potential immune adjuvants and nano carriers. *Int. J. Biol. Macromol. ***154**, 339–348 (2020). 32184144 10.1016/j.ijbiomac.2020.03.065

[61] Sanchaniya, J. V., Lasenko, I., Gobins, V., Kobeissi, A. & Goljandin, D. A finite element Method for determining the Mechanical properties of Electrospun Nanofibrous Mats. *Polym. (Basel)*. **16**, 852 (2024). 10.3390/polym16060852 PMC10974525 38543457

[62] Sheikhi, S., Ghassemi, A., Sajadi, S. M. & Hashemian, M. Comparison of the mechanical characteristics of produced nanofibers by electrospinning process based on different collectors. *Heliyon*. **10**, e23841 (2024). 38205316 10.1016/j.heliyon.2023.e23841 PMC10776987

[63] Augustine, R., Kalarikkal, N. & Thomas, S. Electrospun PCL membranes incorporated with biosynthesized silver nanoparticles as antibacterial wound dressings. *Appl. Nanosci. (Switzerland)*. **6**, 337–344 (2016).

[64] Faraji, S., Nowroozi, N., Nouralishahi, A. & Shabani Shayeh, J. Electrospun poly-caprolactone/graphene oxide/quercetin nanofibrous scaffold for wound dressing: evaluation of biological and structural properties. *Life Sci. ***257**, 118062 (2020). 10.1016/j.lfs.2020.118062 32652138

[65] Fahimirad, S. et al. Wound healing performance of PCL/chitosan based electrospun nanofiber electrosprayed with curcumin loaded chitosan nanoparticles. *Carbohydr. Polym. ***259**, 117640 (2021). 10.1016/j.carbpol.2021.117640 33673981

[66] Shitole, A. A. et al. Poly (vinylpyrrolidone)–iodine engineered poly (ε-caprolactone) nanofibers as potential wound dressing materials. *Mater. Sci. Eng. C. ***110**, 110731 (2020). 10.1016/j.msec.2020.110731 32204042

[67] Chatterjee, A. P. A percolation-based model for the conductivity of nanofiber composites. *J. Chem. Phys. ***139**, 224904 (2013). 10.1063/1.4840098 24329090

[68] Musino, D., Genix, A. C., Chauveau, E., Bizien, T. & Oberdisse, J. Structural identification of percolation of nanoparticles. *Nanoscale*. **12**, 3907–3915 (2020). 32003375 10.1039/c9nr09395h

[69] Vengatesan, M. R., Singh, S., Pillai, V. V. & Mittal, V. Crystallization, mechanical, and fracture behavior of mullite fiber-reinforced polypropylene nanocomposites. *J. Appl. Polym. Sci. ***133**, 43725 (2016).

[70] Li, X., Kanjwal, M. A., Lin, L. & Chronakis, I. S. Electrospun polyvinyl-alcohol nanofibers as oral fast-dissolving delivery system of caffeine and riboflavin. *Colloids Surf., B*. **103**, 182–188 (2013). 10.1016/j.colsurfb.2012.10.016 23201736

[71] Celebioglu, A. & Uyar, T. Fast dissolving oral drug Delivery System based on Electrospun Nanofibrous Webs of Cyclodextrin/Ibuprofen Inclusion Complex Nanofibers. *Mol. Pharm. ***16**, 4387–4398 (2019). 31436100 10.1021/acs.molpharmaceut.9b00798

[72] Pérez, M. et al. Comparison of Antibacterial Activity and Wound Healing in a superficial abrasion mouse model of Staphylococcus aureus skin infection using photodynamic therapy based on Methylene Blue or Mupirocin or both. *Front. Med. ***8**, 673408 (2021). 10.3389/fmed.2021.673408 PMC8185160 34113639

[73] Hiramatsu, K. et al. Multi-drug-resistant Staphylococcus aureus and future chemotherapy. *J. Infect. Chemother. ***20**, 593–601 (2014). 25172776 10.1016/j.jiac.2014.08.001

[74] Gul, A., Gallus, I., Tegginamath, A., Maryska, J. & Yalcinkaya, F. Electrospun Antibacterial nanomaterials for Wound Dressings Applications. *Membranes*. **11**, 908 (2021). 34940410 10.3390/membranes11120908 PMC8707140

[75] Archana, D., Singh, B. K., Dutta, J. & Dutta, P. K. Chitosan-PVP-nano silver oxide wound dressing: in vitro and in vivo evaluation. *Int. J. Biol. Macromol. ***73**, 49–57 (2015). 25450048 10.1016/j.ijbiomac.2014.10.055

[76] Liu, G. et al. Composite membranes from quaternized chitosan reinforced with surface-functionalized PVDF electrospun nanofibers for alkaline direct methanol fuel cells. *J. Membr. Sci. ***611**, 118242–118242 (2020).

[77] Heidari, M., Bahrami, S. H., Ranjbar-Mohammadi, M. & Milan, P. B. Smart Electrospun nanofibers containing PCL/gelatin/graphene oxide for application in nerve tissue engineering. *Mater. Sci. Engineering: C*. **103**, 109768 (2019). 10.1016/j.msec.2019.109768 31349413

[78] Menichetti, A., Mavridi-Printezi, A., Mordini, D. & Montalti, M. Effect of size, shape and surface functionalization on the antibacterial activity of silver nanoparticles. *JFB*. **14**, 244 (2023). 37233354 10.3390/jfb14050244 PMC10219039

[79] Zhou, G. et al. Outer membrane porins contribute to Antimicrobial Resistance in Gram-negative Bacteria. *Microorganisms*. **11**, 1690 (2023). 37512863 10.3390/microorganisms11071690 PMC10385648

[80] Wang, M., Buist, G. & van Dijl, J. M. *Staphylococcus aureus* cell wall maintenance – the multifaceted roles of peptidoglycan hydrolases in bacterial growth, fitness, and virulence. *FEMS Microbiol. Rev. ***46**, fuac025 (2022). 35675307 10.1093/femsre/fuac025 PMC9616470

[81] Li, M. et al. Antibacterial behavior and related mechanisms of martensitic Cu-bearing stainless steel evaluated by a mixed infection model of Escherichia coli and Staphylococcus aureus in vitro. *J. Mater. Sci. Technol. ***62**, 139–147 (2021).

[82] Su, Z. et al. Chitosan/Silver Nanoparticle/Graphene oxide nanocomposites with Multi-drug Release, Antimicrobial, and Photothermal Conversion functions. *Materials*. **14**, 2351 (2021). 33946613 10.3390/ma14092351 PMC8124926

[83] Ardean, C. et al. Factors influencing the antibacterial activity of Chitosan and Chitosan modified by Functionalization. *IJMS*. **22**, 7449 (2021). 34299068 10.3390/ijms22147449 PMC8303267

[84] Chen, Y., Pandit, S., Rahimi, S. & Mijakovic, I. Graphene nanospikes exert bactericidal effect through mechanical damage and oxidative stress. *Carbon*. **218**, 118740 (2024).

[85] More, P. R. et al. Silver nanoparticles: bactericidal and mechanistic Approach against Drug Resistant pathogens. *Microorganisms*. **11**, 369 (2023). 36838334 10.3390/microorganisms11020369 PMC9961011

[86] Swai, E. & Schoonman, L. Microbial quality and associated health risks of raw milk marketed in the Tanga region of Tanzania. *Asian Pac. J. Trop. Biomed. ***1**, 217–222 (2011). 23569762 10.1016/S2221-1691(11)60030-0 PMC3609189

[87] Basiry, D. et al. The effect of disinfectants and antiseptics on co- and cross-selection of resistance to antibiotics in aquatic environments and wastewater treatment plants. *Front. Microbiol. ***13**, 1050558 (2022). 36583052 10.3389/fmicb.2022.1050558 PMC9793094

[88] Gunasekaran, T., Nigusse, T. & Dhanaraju, M. D. Silver nanoparticles as real topical bullets for Wound Healing. *J. Am. Coll. Clin. Wound Spec. ***3**, 82–96 (2011). 24527370 10.1016/j.jcws.2012.05.001 PMC3921230

[89] Bai, D., Zhou, F. & Wu, L. Comparing the efficacy of chlorhexidine and povidone–iodine in preventing surgical site infections: a systematic review and meta-analysis. *Int. Wound J. ***21**, e14463 (2024). 10.1111/iwj.14463 PMC10828524 37885342

[90] Muteeb, G., Rehman, M. T., Shahwan, M. & Aatif, M. Origin of antibiotics and antibiotic resistance, and their impacts on Drug Development: a narrative review. *Pharmaceuticals*. **16**, 1615 (2023). 38004480 10.3390/ph16111615 PMC10675245

[91] Zhu, G., Wang, Q., Lu, S. & Niu, Y. Hydrogen peroxide: a potential wound therapeutic target. *Med. Princ Pract. ***26**, 301–308 (2017). 28384636 10.1159/000475501 PMC5768111

[92] Viscusi, G., Paolella, G., Lamberti, E., Caputo, I. & Gorrasi, G. Quercetin-loaded polycaprolactone-polyvinylpyrrolidone Electrospun membranes for Health Application: design, characterization, modeling and cytotoxicity studies. *Membranes*. **13**, 242 (2023). 36837745 10.3390/membranes13020242 PMC9965405

[93] Shitole, A. A. et al. Poly (vinylpyrrolidone)–iodine engineered poly (ε-caprolactone) nanofibers as potential wound dressing materials. *Mater. Sci. Engineering: C*. **110**, 110731 (2020). 10.1016/j.msec.2020.110731 32204042

[94] Liang, H. et al. Engineering Multifunctional films based on metal-phenolic networks for rational pH-Responsive delivery and cell imaging. *ACS Biomaterials Sci. Eng. ***2**, 317–325 (2016). 10.1021/acsbiomaterials.5b00363 33429535

[95] Quinteros, M. A., Cano Aristizábal, V., Dalmasso, P. R., Paraje, M. G. & Páez, P. L. Oxidative stress generation of silver nanoparticles in three bacterial genera and its relationship with the antimicrobial activity. *Toxicol. In Vitro*. **36**, 216–223 (2016). 27530963 10.1016/j.tiv.2016.08.007

[96] Sorinolu, A. J., Godakhindi, V., Siano, P., Vivero-Escoto, J. L. & Munir, M. Influence of silver ion release on the inactivation of antibiotic resistant bacteria using light-activated silver nanoparticles. *Mater. Adv. ***3**, 9090–9102 (2022). 36545324 10.1039/d2ma00711h PMC9743134

[97] Lin, L. et al. Membrane-disruptive peptides/peptidomimetics-based therapeutics: promising systems to combat bacteria and cancer in the drug-resistant era. *Acta Pharm. Sinica B*. **11**, 2609–2644 (2021). 10.1016/j.apsb.2021.07.014 PMC8463292 34589385

[98] Xiaoli, F. et al. Graphene oxide disrupted mitochondrial homeostasis through inducing intracellular redox deviation and autophagy-lysosomal network dysfunction in SH-SY5Y cells. *J. Hazard. Mater. ***416**, 126158 (2021). 34492938 10.1016/j.jhazmat.2021.126158

[99] Xuan, L., Ju, Z., Skonieczna, M., Zhou, P. & Huang, R. Nanoparticles-induced potential toxicity on human health: Applications, toxicity mechanisms, and evaluation models. *MedComm* **4**, e327 (2023). 37457660 10.1002/mco2.327 PMC10349198

[100] Carrasco-Torres, G. et al. Cytotoxicity, Oxidative Stress, Cell Cycle Arrest, and Mitochondrial Apoptosis after Combined Treatment of Hepatocarcinoma Cells with Maleic Anhydride Derivatives and Quercetin. *Oxidative Med Cell Longevity* **2017**, 2734976 (2017). 10.1155/2017/2734976 PMC5661749 29163752

[101] Farabi, B. et al. The efficacy of stem cells in Wound Healing: a systematic review. *IJMS*. **25**, 3006 (2024). 38474251 10.3390/ijms25053006 PMC10931571

[102] Floroian, L. & Badea, M. Vivo Biocompatibility Study on Functional nanostructures containing bioactive glass and plant extracts for Implantology. *IJMS*. **25**, 4249 (2024). 38673834 10.3390/ijms25084249 PMC11050673

