# Supplementary material for: Integrating an antimicrobial nanocomposite to bioactive electrospun fibers for improved wound dressing materials
Source: Sci Rep. 2024 Oct 24;14:25118. doi: 10.1038/s41598-024-75814-2 (PMC11499993; doi:10.1038/s41598-024-75814-2)
Supplement: Supplementary file 1 — Supplementary Material 1. [file 41598_2024_75814_MOESM1_ESM.docx]

Supplementary Information

**Integrating Chitosan, Silver Nanocrystals, and Graphene Oxide (ChAgG) into Electrospun PCL/PVP Fibers for Improved Wound Dressings. Part II.**

Victoria Leonor Reyes Guzmán^1^, Luis Jesús Villarreal Gómez^*1,2^, Rubi Vázquez Mora^3^, Yesica Itzel Méndez Ramírez^3^, Juan Antonio Paz González^1^, Arturo Zizumbo López^4^, Hugo Borbón^5^, Eder Germán Lizarraga Medina^1^, José Manuel Cornejo Bravo^2^, Graciela Lizeth Pérez González^1^, Arturo Sinue Ontiveros Zepeda^6^, Armando Pérez Sánchez^1^, Elizabeth Chavira-Martínez^7^, Rafael Huirache-Acuña^8^, Yoxkin Estévez-Martínez^3*^

^1^Facultad de Ciencias de la Ingeniería y Tecnología, Universidad Autónoma de Baja California, Blvd. Universitario #1000. Unidad Valle de las Palmas. Tijuana, Baja. CP. 21500, Tijuana, Baja California, México

^2^Facultad de Ciencias Química e Ingeniería, Universidad Autónoma de Baja California, Universidad #14418, UABC, Parque Internacional Industrial Tijuana, 22424, Tijuana, Baja California, México

^3^Tecnológico Nacional de México, Campús Acatlán de Osorio, Carretera Acatlán - San Juan Ixcaquistla kilómetro 5.5, Del Maestro, Unidad Tecnológica Acatlán, Acatlán, Puebla. 74949, México.

^4^Tecnológico Nacional de México, Campus Tijuana, Blvd. Alberto Limón Padilla y Av. ITR

Tijuana S/N, Colonia Mesa de Otay C.P. 22500 Tijuana, Baja California, México

^5^Centro de Nanociencias y Nanotecnología, Universidad Nacional Autónoma de México, Carr. Tijuana-Ensenada km107, C.I.C.E.S.E., 22860, Ensenada, Baja California, México

^6^Facultad de Ciencias de la Ingeniería, Administrativas y Sociales, Universidad Autónoma de Baja California, Blvrd Universidad 1, San Fernando, 21460 Tecate, B.C.

^7^Instituto de Investigaciones en Materiales, Circuito Exterior S/N Circuito de la, Investigación Científica, C.U., 04510 Ciudad de México, México.

^8^Facultad de Ingeniería Química, Universidad Michoacana de San Nicolás de Hidalgo, Michoacán, Morelia, 58060, Mexico

*Corresponding author: yoxkin@gmail.com and luis.villarreal@uabc.edu.mx.


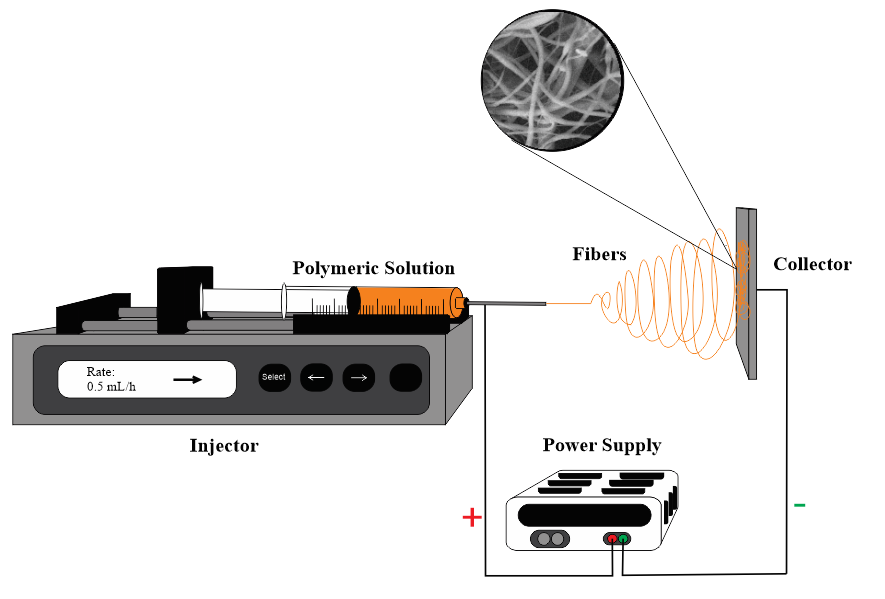


**Figure-S1.** Electrospinning setup.


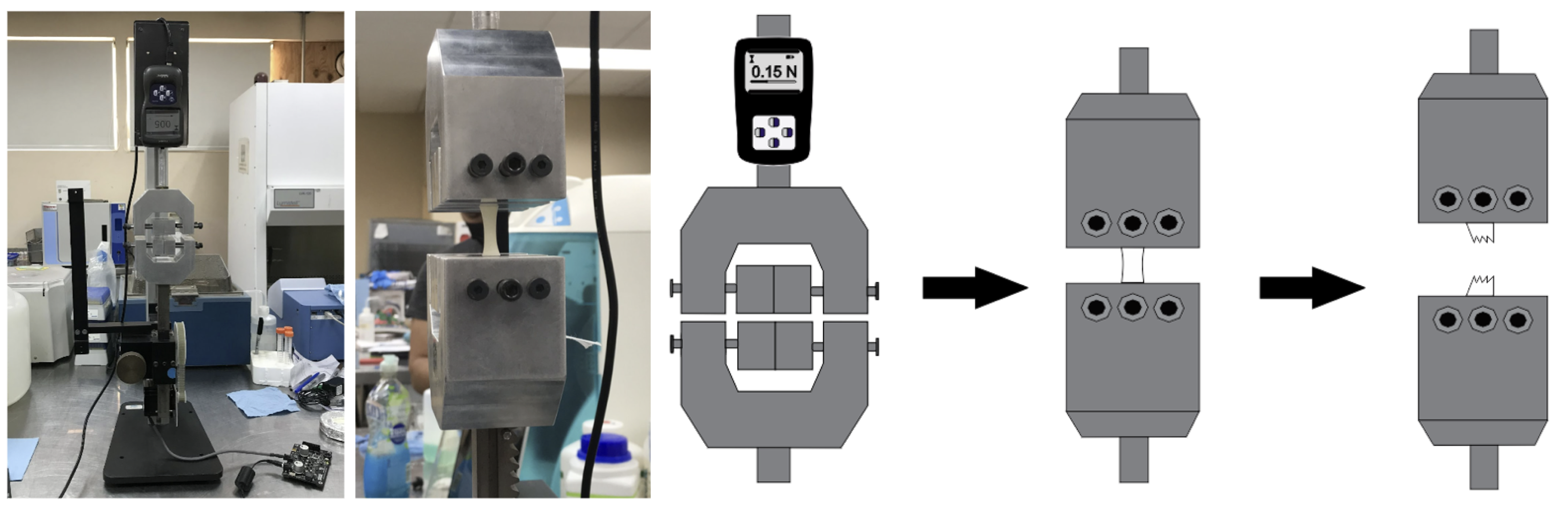


**Figure-S2.** Mechanical testing of the electrospun fibers according to the ASTM D638 standard.


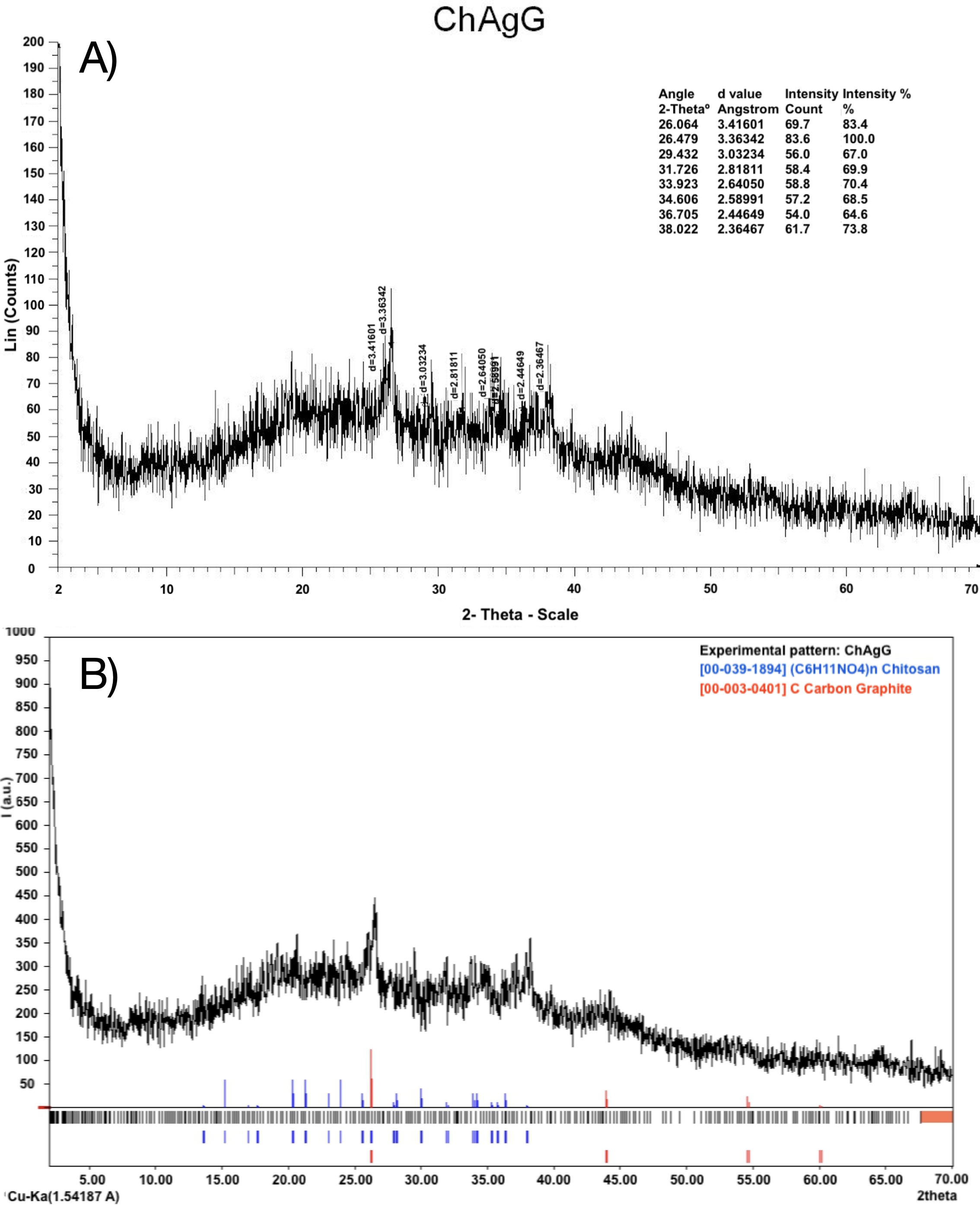


**Figure-S3.** XRD for the sample corresponding to *ChAgG* nanocomposite.


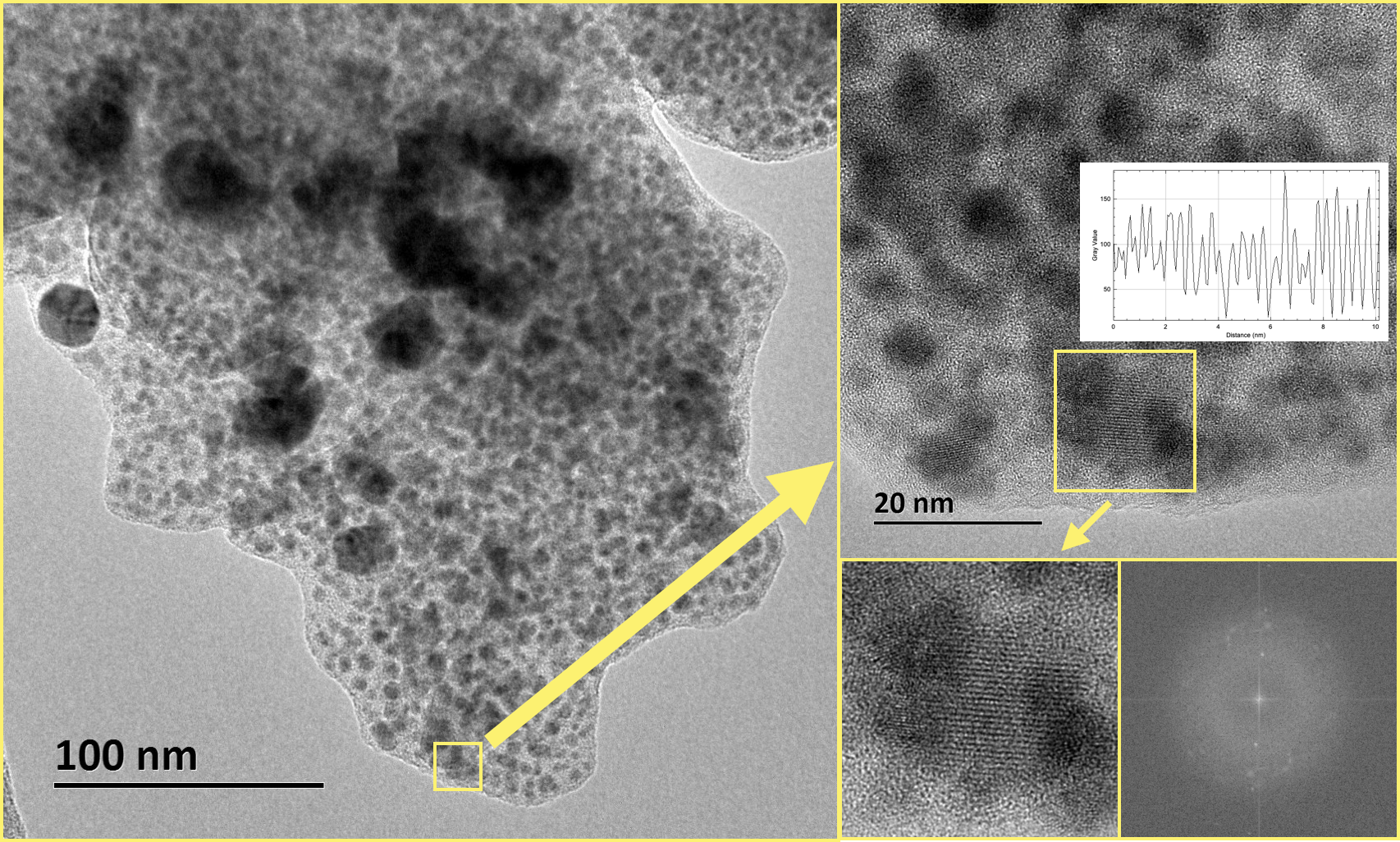


**Figure-S4.** High-resolution transmission electron micrographs for *ChAgG.*


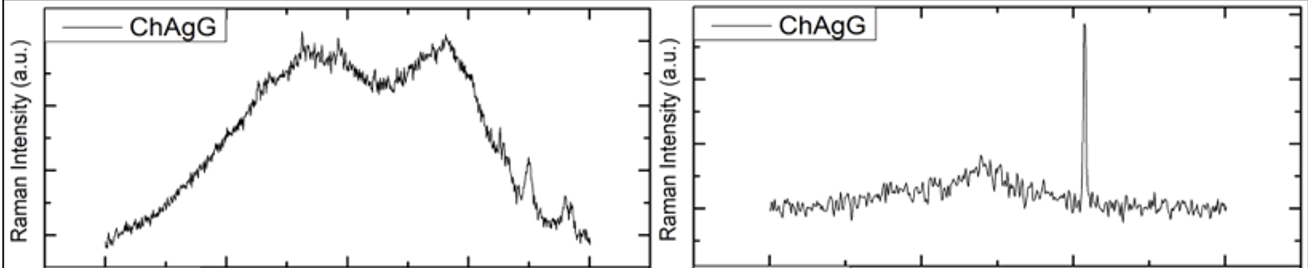

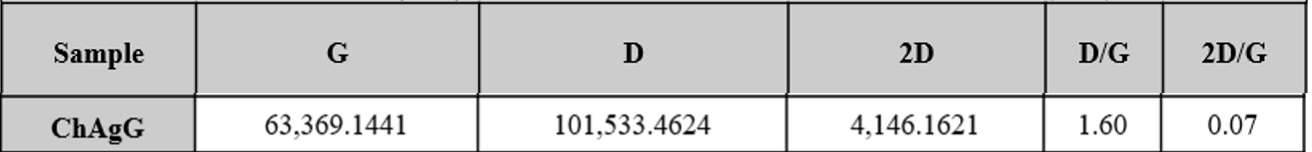


**Figure-S5.** The Raman spectroscopy was performed on Graphene (Gr), Oxidized Graphene (GrOx), and the nanocomposite (ChAgG).


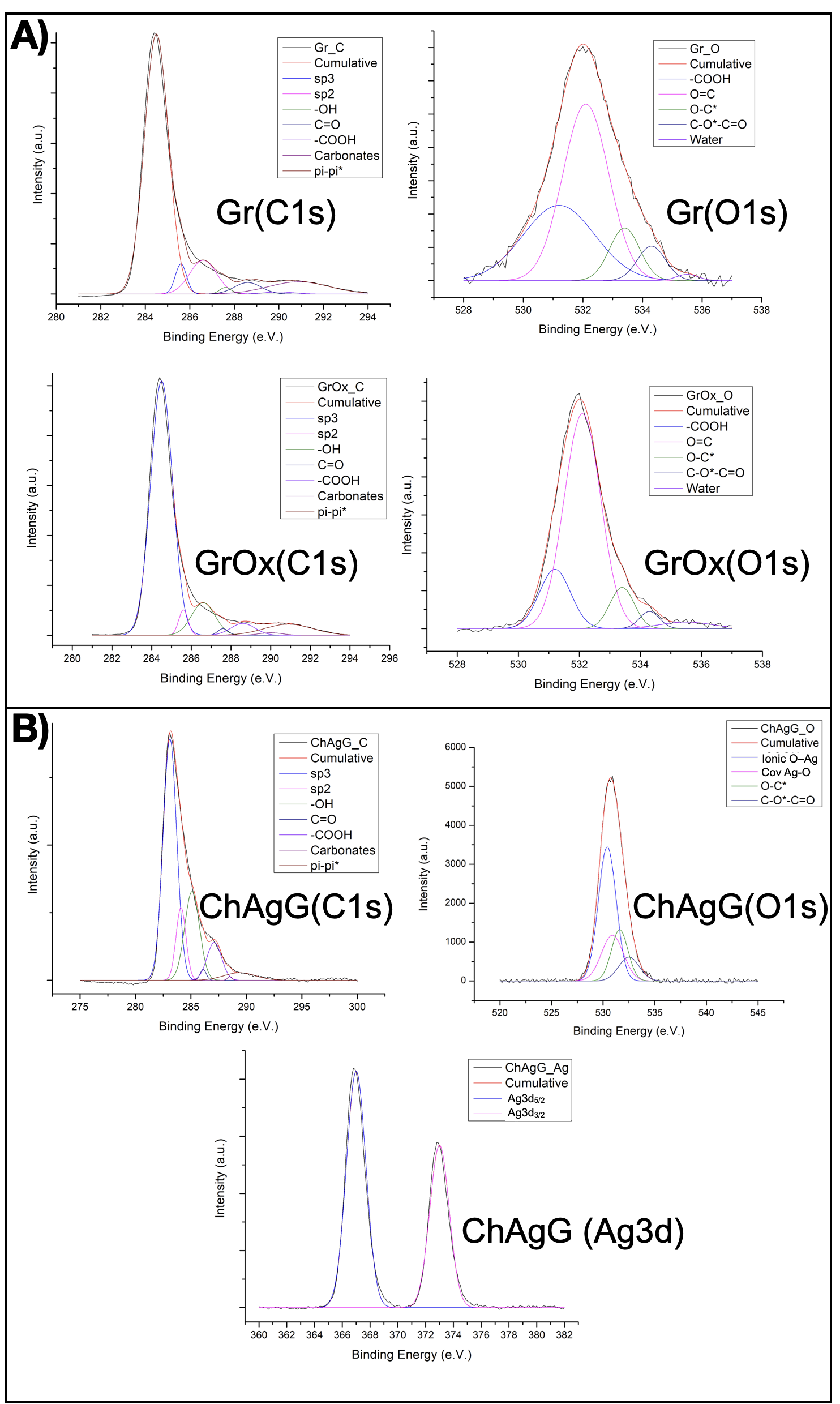


**Figure-S6.** A) XPS deconvolution of C1s and O1s of the Gr and GrOx. B) XPS deconvolution of C1s and O1s of the Nanocomposite ChAgG (Ag3d).


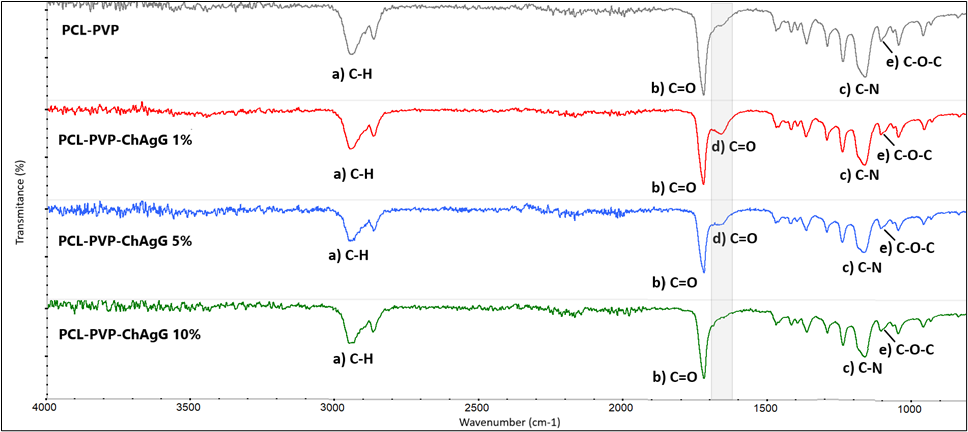


**Figure-S7.** FTIR analysis of the *PCL/PVP* and *PCL/PVP-ChAgG* electrospun fibers


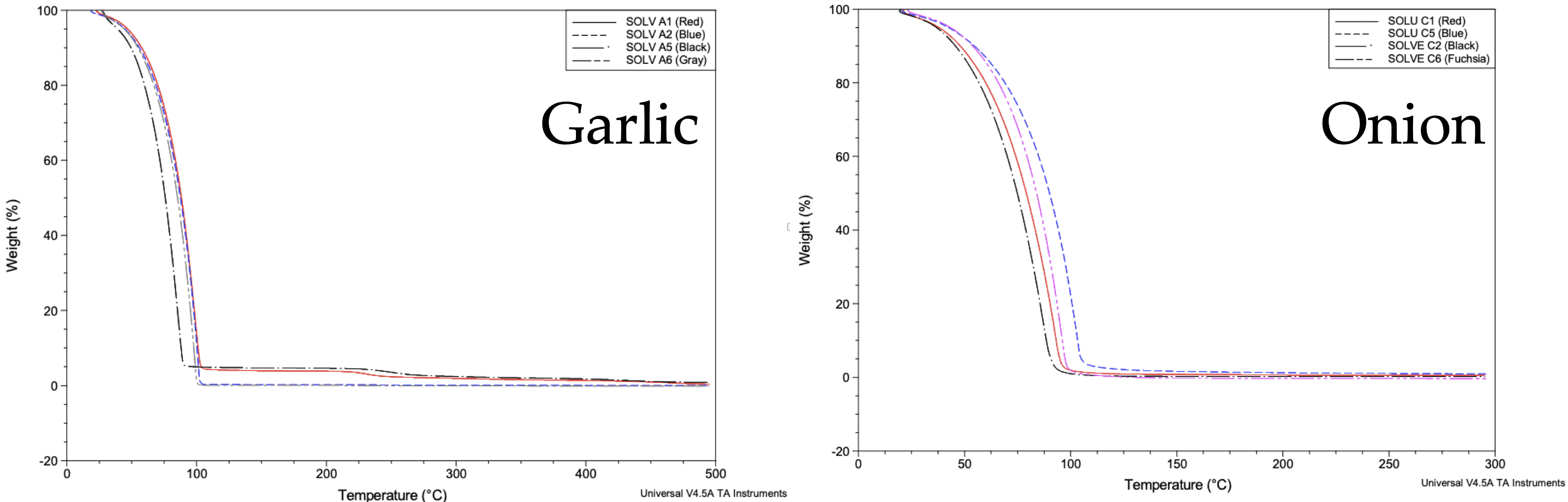


**Figure-S8.** Thermograms of garlic silver nanocrystals as precursors.

**
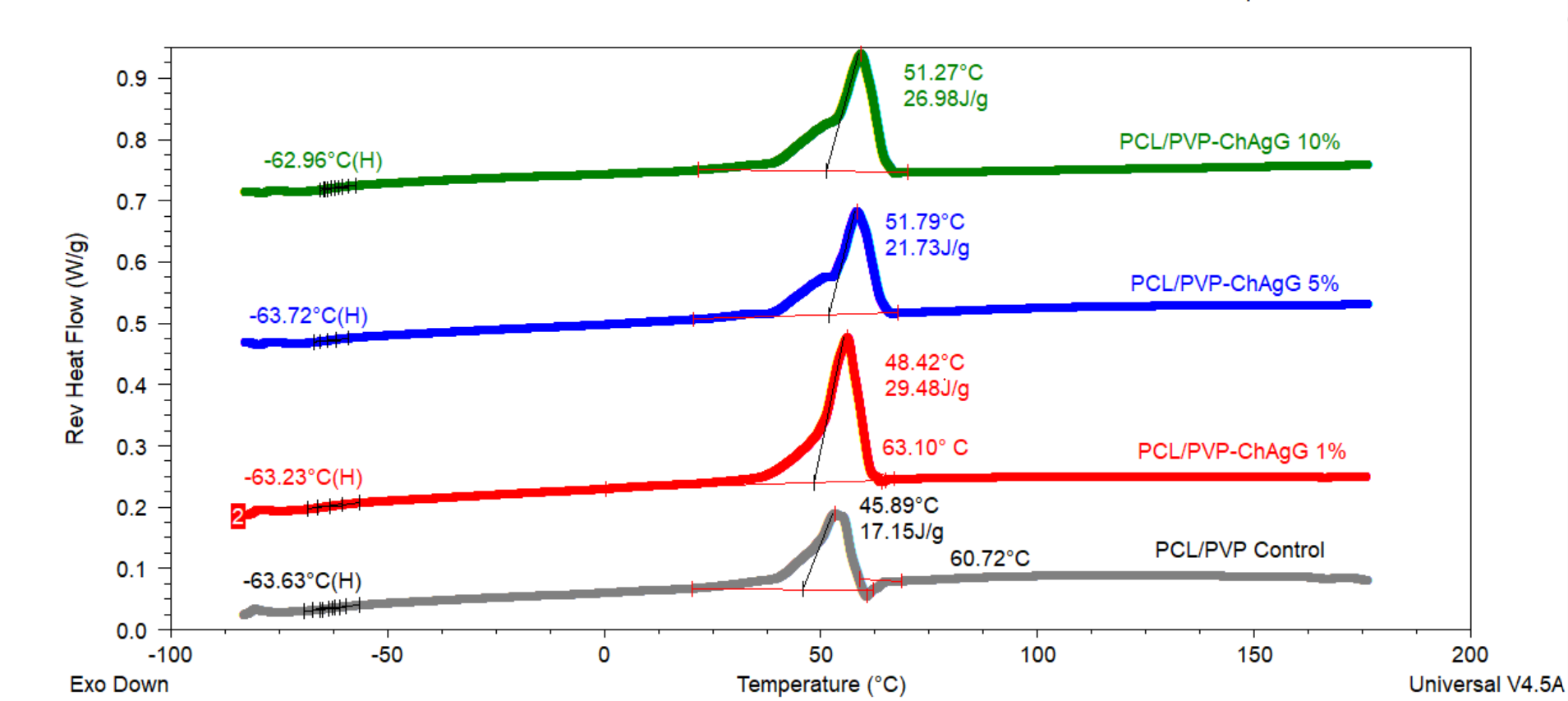
**


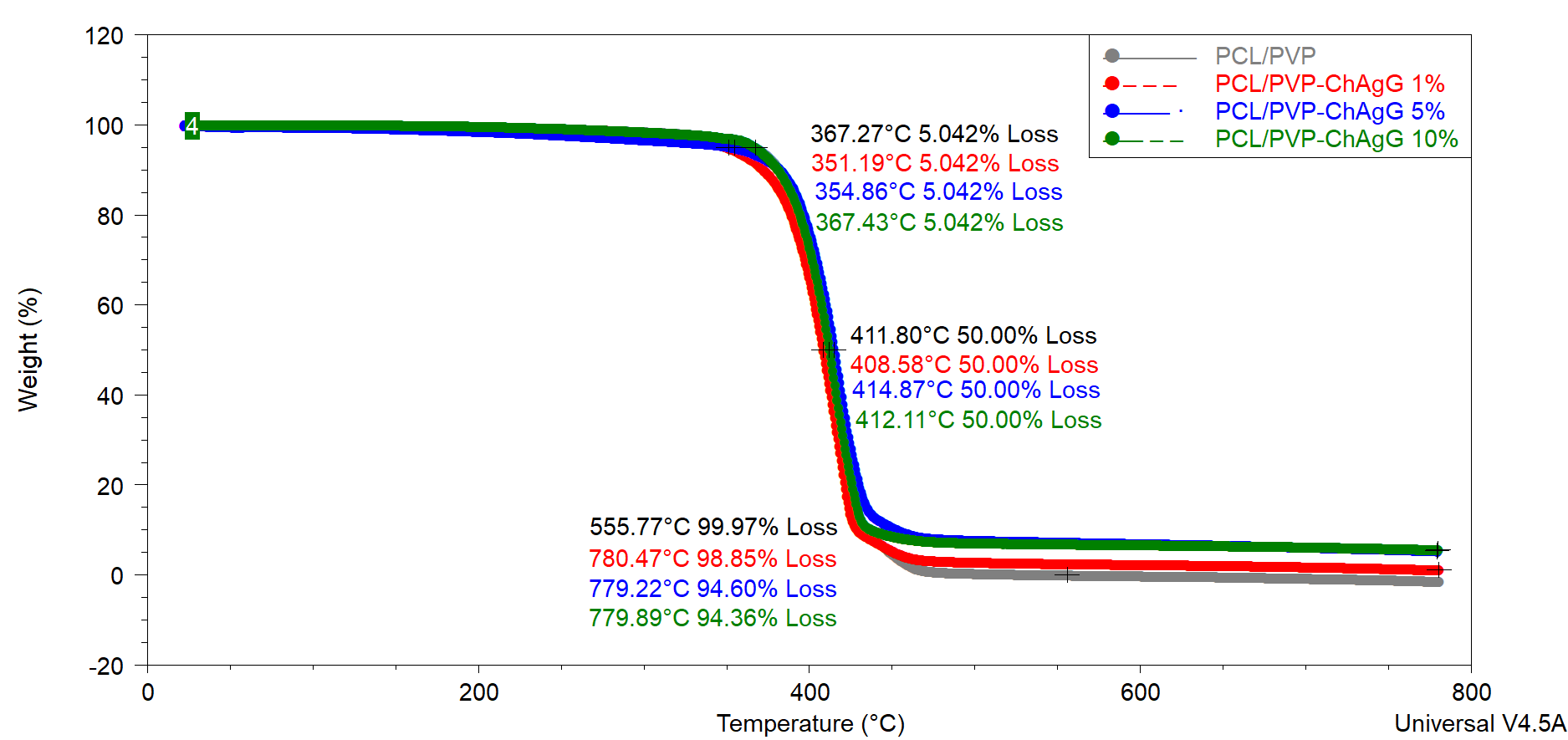


**Figure-S9.** DSC and TGA analysis of the *PCL/PVP* and *PCL/PVP-ChAgG* electrospun fibers. Above figure correspond to the DSC graph, below figure correspond to TGA graph.


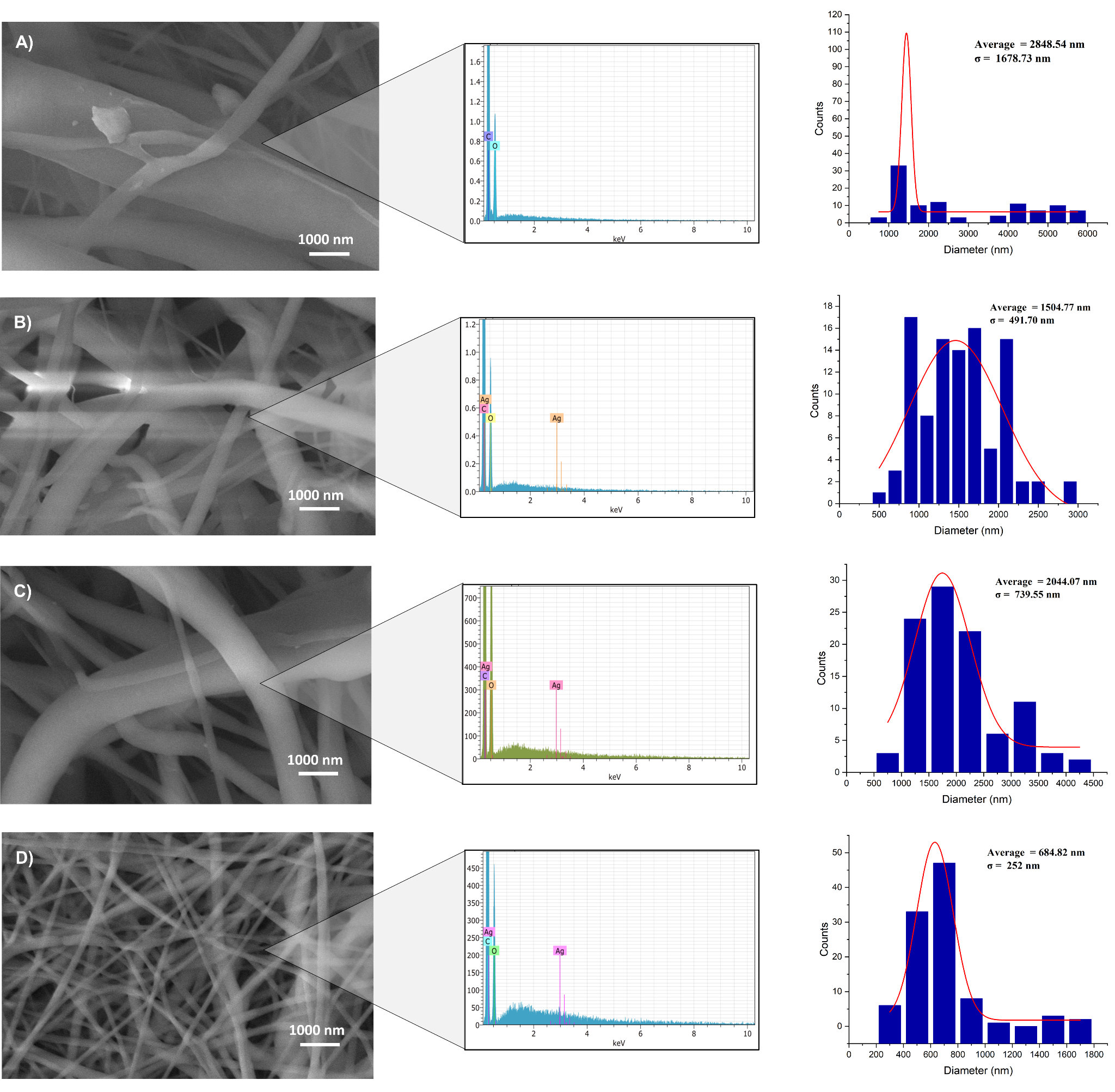


**Figure-S10.** EDX analysis *PCL/PVP* and *PCL/PVP-ChAgG* fibers. A) *PCL/PVP* Control. B) *PCL/PVP-ChAgG* 5%. C) *PCL/PVP-ChAgG* 10%.
